# *DeNovo* Amyloid Peptide–Polymer Blends with Enhanced Mechanical and Biological Properties

**DOI:** 10.1021/acsapm.4c04020

**Published:** 2025-03-12

**Authors:** Xianjun Wang, Malay Mondal, Penelope E. Jankoski, Lisa K. Kemp, Tristan D. Clemons, Vijayaraghavan Rangachari, Sarah E. Morgan

**Affiliations:** 1 School of Polymer Science and Engineering, University of Southern Mississippi, Hattiesburg, Mississippi 39406, United States; 2 Department of Chemistry and Biochemistry, School of Mathematics and Natural Sciences, University of Southern Mississippi, Hattiesburg, Mississippi 39406, United States; 3 Center for Molecular and Cellular Biosciences, University of Southern Mississippi, Hattiesburg, Mississippi 39406, United States

**Keywords:** amyloid, self-assembly, peptide−polymer blend, electrospinning, composite nanofibers

## Abstract

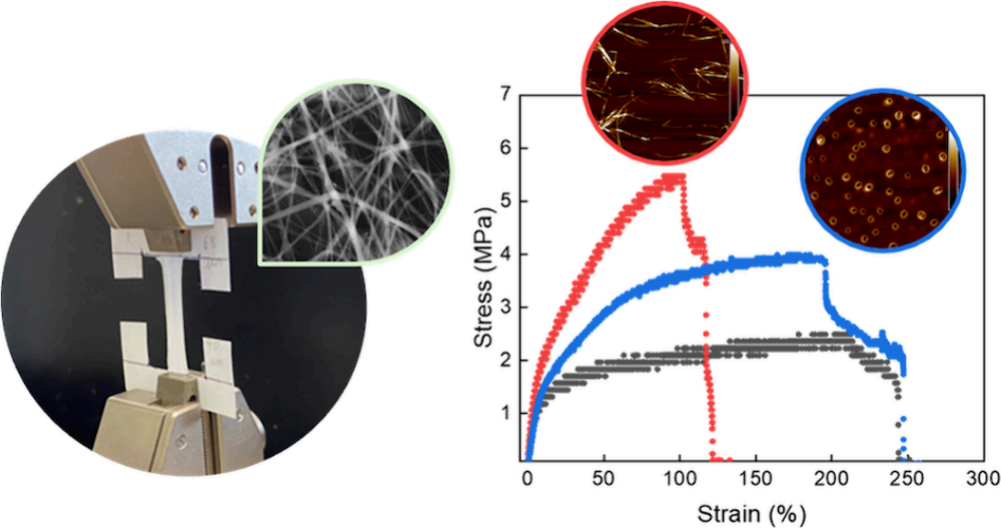

Amyloid peptides are structurally diverse materials that exhibit different properties depending on their self-assembly. While they are often associated with neurodegenerative diseases, functional amyloids play important roles in nature and exhibit properties with high relevance for biomedical applications, including remarkable strength, mechanical stability, antimicrobial and antioxidant properties, low cytotoxicity, and adhesion to biotic and abiotic surfaces. Challenges in developing amyloid biomaterials include the complexity of peptide chemistry and the practical techniques required for processing amyloids into bulk materials. In this work, two *de novo* decapeptides with fibrillar and globular morphologies were synthesized, blended with poly(ethylene oxide), and fabricated into composite mats via electrospinning. Notable enhancements in the mechanical properties of the composite mats were observed, attributed to the uniform distribution of the peptide assemblies within the PEO matrix and interactions between the materials. Morphological differences, such as the production of thinner nanofibers, are attributed to the increased conductivity from the zwitterionic nature of the decapeptides. Blend rheology and postprocessing analysis revealed how processing might affect the amyloid aggregation and secondary structure of the peptides. Both decapeptides demonstrated low cytotoxicity and strong antioxidant activity, indicating their potential for safe and effective use as biomaterials. This research lays the foundation for designing amyloid peptides for specific applications by defining the structure–property-processing relationships of the *de novo* peptide–polymer blends.

## Introduction

Protein aggregates called amyloids, commonly associated with pathologies such as Alzheimer’s disease, Parkinson’s disease, and spongiform encephalopathies, have been found to form insoluble deposits and hydrogels at elevated concentrations due to hierarchical self-assembly into large, entangled fiber networks.^1^ Emerging research has uncovered nonpathological roles for amyloids in cellular systems, highlighting their unique mechanical stability and biochemical properties.^2−6^ Amyloid functions are associated with their cross β-sheet structure in which parallel β-sheets are stacked perpendicular to the fibril axis through hydrophobic interactions, and hydrogen bonds run parallel to the fibril axis.^7^ Amyloids are structurally versatile materials that exhibit diverse properties through their self-assembled structures. For instance, amyloids have been shown to play crucial roles in biofilm formation by creating a protective extracellular matrix, which allows bacterial colonies to thrive in different environments.^8−10^ While amyloid matrices provide a protective environment by enhancing cell adhesion and shielding microbes from external threats, this structural integrity also facilitates their antimicrobial function. The same cross β-sheet structure that reinforces microbial biofilms and provides mechanical stability also enables amyloid peptides to entrap or disrupt the membranes of competing microorganisms, thereby exerting antimicrobial effects.^4,11^ The nature of this activity depends on the specific bacterial interactions: in some cases, amyloid peptides promote biofilm formation and adhesion, while in others, they function as antimicrobial agents through direct membrane penetration or entrapment of pathogens.^1,11,12^ Amyloid peptides can also cause cell membrane disruption, which results in broad-spectrum antimicrobial properties.^13^ Moreover, amyloids can agglutinate microbial cells, further enhancing their antimicrobial effectiveness by immobilizing and neutralizing pathogens.^11^ These examples showcase the versatility of self-assembled cross β-sheet structures.

The amyloids CsgA from *E. coli* and FapC from *Pseudomonas* illustrate the diversity of amyloid structure and function. CsgA is the major amyloid-forming protein encoded within the curli operon in *E. coli*. CsgA undergoes self-assembly to form β-sheet-rich amyloid fibrils, the structural components of curli fibers found in extracellular biofilms, which provide structural integrity and enable bacterial adhesion.^8,14^ FapC is the major subunit of the functional amyloid protein (Fap) system, which helps assemble amyloid fibrils that stabilize the biofilm matrix. These fibrils are essential for maintaining biofilm cohesion and promoting bacterial survival under stressful conditions. Unlike CsgA, FapC is prone to form disordered amyloid fibrils. This allows *Pseudomonas* biofilms to exhibit flexibility in their structural properties, adjusting to different environmental conditions.^9,10^ The protein also plays a role in biofilm dispersal, essential for bacterial colonization of new surfaces.

Other than cell-to-cell adhesion^15^ and cell-to-host adhesion,^14^ some functional amyloids and their synthetic analogs adhere strongly to abiotic surfaces.^16−19^ The strength of a single amyloid fibril was reported to be 0.6 ± 0.4 GPa, the same order of magnitude as the strength of steel (0.6–1.8 GPa).^4,20^ Some plant-derived amyloids, including those from rice, whey, and soy proteins, have demonstrated antioxidant activity.^21−23^ This property may originate from oxidizable amino acid residues, and research also suggests the fibrillization can affect the antioxidant activity.^24^ These diverse properties show the attractiveness of functional amyloids as building blocks for protein-based functional materials such as drug-releasing vehicles,^25^ tissue engineering,^26−28^ filtration devices to remove contaminants,^29,30^ biosensors,^31,32^ and bioplastics.^33−35^

A practical aspect of using amyloids as functional materials is developing feasible solution-based processing techniques that retain the desired morphology and properties while removing the solvent. For instance, Li et al. harvested amyloid proteins from plants, partially denatured them, allowed them to self-assemble, and then solution-cast them into thin films. This process led to uncontrolled amyloid nanofibril formation, affecting the transparency of the resulting Bioplastic films due to the sensitivity of amyloid morphology to environmental conditions.^33^ Peydayesh et al. fabricated amyloid fibril/poly(vinyl alcohol) (PVA) blends into free-standing, transparent, and flexible Bioplastic films for packaging applications with superior sustainability.^34^ Bagnani et al. investigated mixtures of amyloids extracted from rapeseed cake with methylcellulose and glycerol and reported that the optimized biodegradable plastic blend film was very ductile.^35^ Both works mentioned that the amyloid assembly state (i.e., monomeric or fibril) affects the mechanical properties of composite films. Electrospinning is another popular solution-processing technique for developing biomaterials. It has been demonstrated that some peptides can be directly electrospun into fibers; however, this generally requires high concentrations and fairly long amino acid sequences,^36^ and it is often desirable to employ a carrier polymer for specific applications. Initial reports have explored the electrospinning of fibril-forming peptide/polymer blends, and the results vary widely. To our knowledge, specific studies of designed ***de novo*** amyloid peptides and their structure/property/processing relationships are lacking. Brun et al. prepared electrospun solutions of peptides with different self-assembling sequences in PEO to form scaffolds and reported higher cell adhesion for materials enriched with β-hairpin or β-sheet peptides,^37^ but did not report mechanical properties of the fibers. Rubin et al. reported the preparation of poly(D,L-lactic acid) PDLLA electrospun nanofibers reinforced with self-assembling D,L-cyclic peptides, and found that higher nanofiber peptide incorporation resulted in stronger single fibers.^38^ Kauer et al. synthesized a self-assembling folic acid conjugated dipeptide, folate l-tyrosyl-l-tyrosine methyl ester, and prepared electrospun fiber mats from solution blends with polycaprolactone. They reported increased tensile stress for mats containing peptide fibrils, but mechanical properties decreased with increased peptide loading.^39^ Kim et al. demonstrated that designed peptides could be assembled to form rigid rod polymers, which when electrospun with PEO at high concentrations formed high modulus fibers.^40^ The different findings reported for these diverse systems highlight the need for understanding the relationship between peptide sequence and processing on the self-assembly process because the resulting tertiary structures influence the potential interactions between the amyloids and polymer matrix.

Despite advancements in the field of amyloids, understanding the relationship between amyloid sequences, intrinsic self-assembling morphologies, and varied processing strategies for fabricating functional amyloid-based biomaterials remains elusive. The primary challenges include the complex nature of amyloid chemistry and the need for a compatible polymer/solvent system to facilitate amyloid assembly. In our previous publication, we specifically examined a library of *de novo* synthetic decapeptides (DPs) containing an amyloidogenic core.^41^ We systematically varied single amino acid residues in two positions to demonstrate the effects of polarity, hydrophobicity, and charge individually.^41^ Building on this work, we have selected two decapeptides, DP I and DP II, with well-defined sequences and distinct assembly morphologies for evaluation in the electrospinning of composite nanofiber mats. DP I is considered a peptide amphiphile, with two hydrophilic ends (serine and aspartic acid at the C-terminus, and lysine at the N-terminus) and a hydrophobic central sequence. Pure DP I forms stable β-sheets and assembles into fibril-like structures, which change to a β-turn conformation at elevated temperatures. DP II also shows overall amphiphilicity, with a distribution of hydrophobic amino acids (alanine and methionine) among hydrophilic amino acids along the chain. Pure DP II exhibits a globular morphology, but its detailed conformation was not fully explored in our previous report.^41^ The theoretical isoelectric point (pI) for DP I is around 7.0, where DP I has five residues for protonation or deprotonation. DP II has a theoretical pI at 9.9 with nine potentially charged residues. Pure DPs have poor electrospinnability, and it is necessary to blend them with an appropriate carrier polymer. Poly(ethylene oxide) (PEO) was chosen in this study because it is nontoxic, highly electrospinnable, and has good solubility in the peptide assembly solvent mixture (ethanol/water). PEO is widely used in biomedical and personal care applications, and it is an appropriate carrier for applications such as wound dressings and skin treatments, where designed amyloids hold the promise of imparting enhanced mechanical strength, antibiotic, and antioxidant properties.^42−44^ Different DP/PEO blends were prepared and fabricated into composite mats via electrospinning. The aim of this comparative research effort is to determine the effects of decapeptide sequence, assembly morphology, processing, and loading level on the thermal, mechanical, morphological, and antioxidant properties of composite nanofiber mats. This study lays the foundation for designing new and customizable amyloid-based biomaterials.

## Results and Discussion

### Secondary Structure and Morphology of Self-Assembled Peptide Aggregates

**Figure 1:**
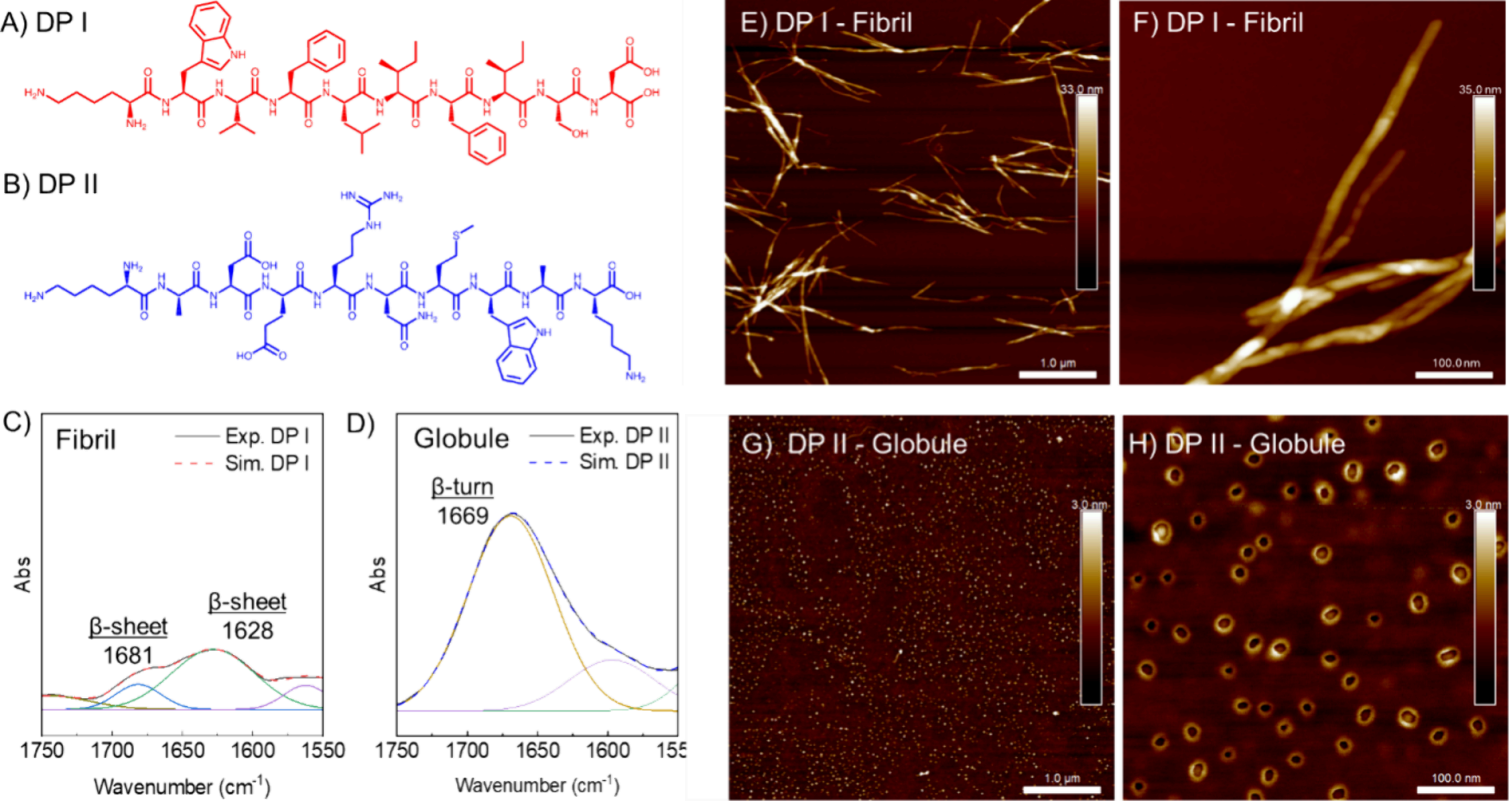
(A, B) Structure of DP I and DP II. (C, D) Deconvoluted amide I region of DPs reveals distinct secondary structures, where DP I shows β-sheet character and DP II shows β-turn character. The solid line represents experimental data and the dashed line is the fitted result based on peak deconvolution. (E–H) AFM height images show long fibril-like structures for DPI and globular structures for DP II.

Two *de novo* peptides, DP I and DP II (Figures 1A and B), were synthesized as described in the experimental section, with crude peptides used without further purification. The purities of DP I and DP II were approximately 75% and 90%, respectively, estimated from LC-MS (Figure S1 and S2). The two peptide structures were selected due to their propensity to form fibril (DP I) and globular (DP II) morphologies in their purified form, as reported previously.^41^ FTIR spectra of the crude DPs in the range of 1750–1550 cm^–1^ are presented in Figure 1 C–D, and full spectra are shown in Figure S3. A deuterated solvent mixture was used in this experiment to specifically suppress the strong water bending mode that overlaps in the Amide I region (1700–1600 cm^–1^), enabling better background subtraction. DP I shows two distinct peaks at 1628 and 1681 cm^–1^, suggesting a mixture of parallel and antiparallel β-sheets, while DP II displays a single peak at 1669 cm^–1^, indicating predominantly β-turns.^45,46^ These findings are consistent with our previous reports.^41^ AFM images of assembled DP I and DP II are presented in Figure 1 E-H and Figure S10. DP I fibrils reveal widths of ∼20 nm and lengths of up to several micrometers, similar to those observed previously.^41^ DP II forms globular structures with diameters of 15–20 nm, which supports the FTIR data on the lack of β-sheet necessary to form fibrils.

### Morphology of Composite Mats

**Figure 2:**
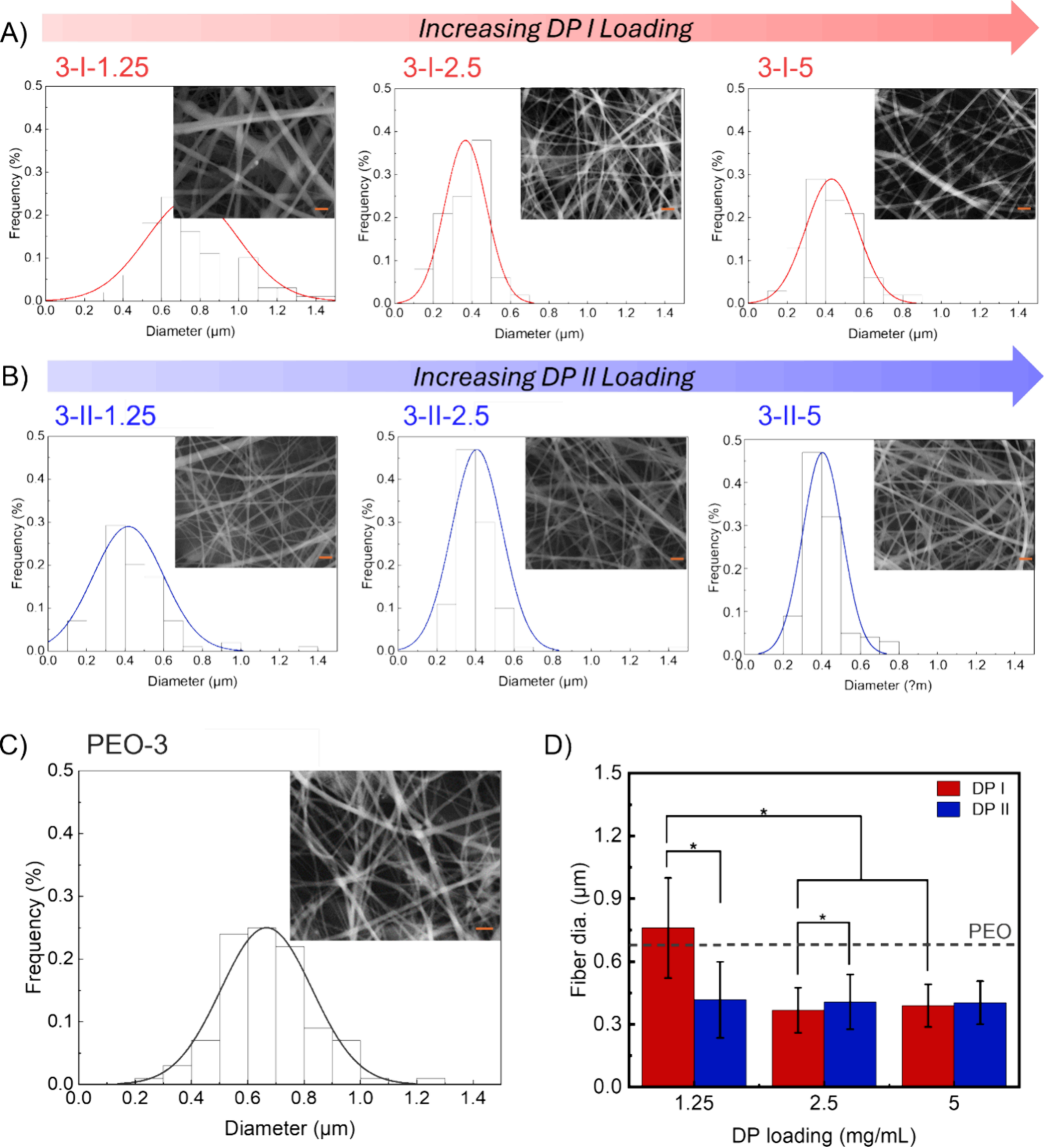
(A) Fiber diameter distribution plots of PEO/DP I composite mats. DP I loading level significantly affects the fiber diameter. (B) Fiber diameter distribution plots of PEO/DP II composite mats. DP II loading level does not affect the fiber diameter. (C) Fiber diameter distribution plots of PEO-3 (the control). (D) Fiber diameter plot for electrospun mats. The dashed line represents the control value (PEO-3); * denotes a statistically significant mean difference at *p* < 0.05. All scale bars = 2 μm.

Solution blends of PEO with varying loadings of DP were prepared and electrospun as described in the experimental section (Table 4). The blends are abbreviated as wt %PEO–DPtype-DP concentration, indicating sample 3-I-1.25 consists of 3 wt % PEO with DP I at a concentration of 1.25 mg/mL. After successful electrospinning, the morphology of the composite mats was characterized using a scanning electron microscope (SEM) (Figure 2), and a detailed fiber diameter analysis is reported in Table 1. Under identical processing conditions, most composite mats display significantly thinner fibers compared to the control (PEO-3), with the exception of sample 3-I-1.25. Increasing DP I incorporation leads to a substantial reduction in fiber diameter, and at higher DP I loading levels (2.50 and 5.00 mg/mL), the composite mats exhibit a much narrower fiber diameter distribution. The loading level of DP II does not affect the mean fiber diameter but narrows the distribution. The observed reduction in fiber diameter and narrowing of the fiber diameter distribution in composite mats may be attributed to shear thinning viscosity effects at electrospinning shear rates (described in the viscosity section) and the zwitterionic nature of DP I and DP II, which alters the conductivity of the polymer/peptide blend. A common strategy to tune the morphology of nanofibers is to add salts to increase the conductivity of the electrospinning solution.^47,48^ The conductivity of the blank solvent mixture (ethanol/water = 3:1, v/v) is 2.1 ± 0.1 μS/cm. However, the conductivity of the DP solutions increases dramatically, with DP I showing 78.8 ± 0.2 μS/cm and DP II showing 111.2 ± 0.2 μS/cm at a concentration of 1.25 mg/mL (Table Supporting Information). Given the intrinsic zwitterionic character of DPs, they enhance the charge density on the surface of the ejected jet during spinning, resulting in higher elongation forces under the electric field. This increase in charge density enhances the overall tension in the fibers due to the self-repulsion of the excess charges on the jet. Consequently, as the charge density increases, the jets become smaller and more spindle-like, leading to a substantial reduction in the fiber diameter.^49^ The conductivity of the 3-I-1.25 solution is the lowest, and this composition produces the highest diameter fibers (0.76 μm). At higher DP-I loadings the conductivity is increased, and the mean fiber diameter is comparable across all fibril solutions (0.4 ±0.03), indicating that there may be a concentration-dependent threshold conductivity at which diameter is decreased.

**Table 1:** Morphology of PEO/DP Composite Mats

	fiber diameter (μm)		
sample ID	mean ± SD (*n* = 100)	max	min
PEO-3	0.67 ± 0.16	1.26	0.29
3-I-1.25	0.76 ± 0.24	1.56	0.34
3-I-2.5	0.37 ± 0.11	0.68	0.17
3-I-5	0.39 ± 0.10	0.66	0.17
3-II-1.25	0.42 ± 0.18	1.33	0.08
3-II-2.5	0.41 ± 0.13	1.40	0.24
3-II-5	0.40 ± 0.10	0.75	0.22

### Tensile Properties of Composite Mats

**Figure 3:**
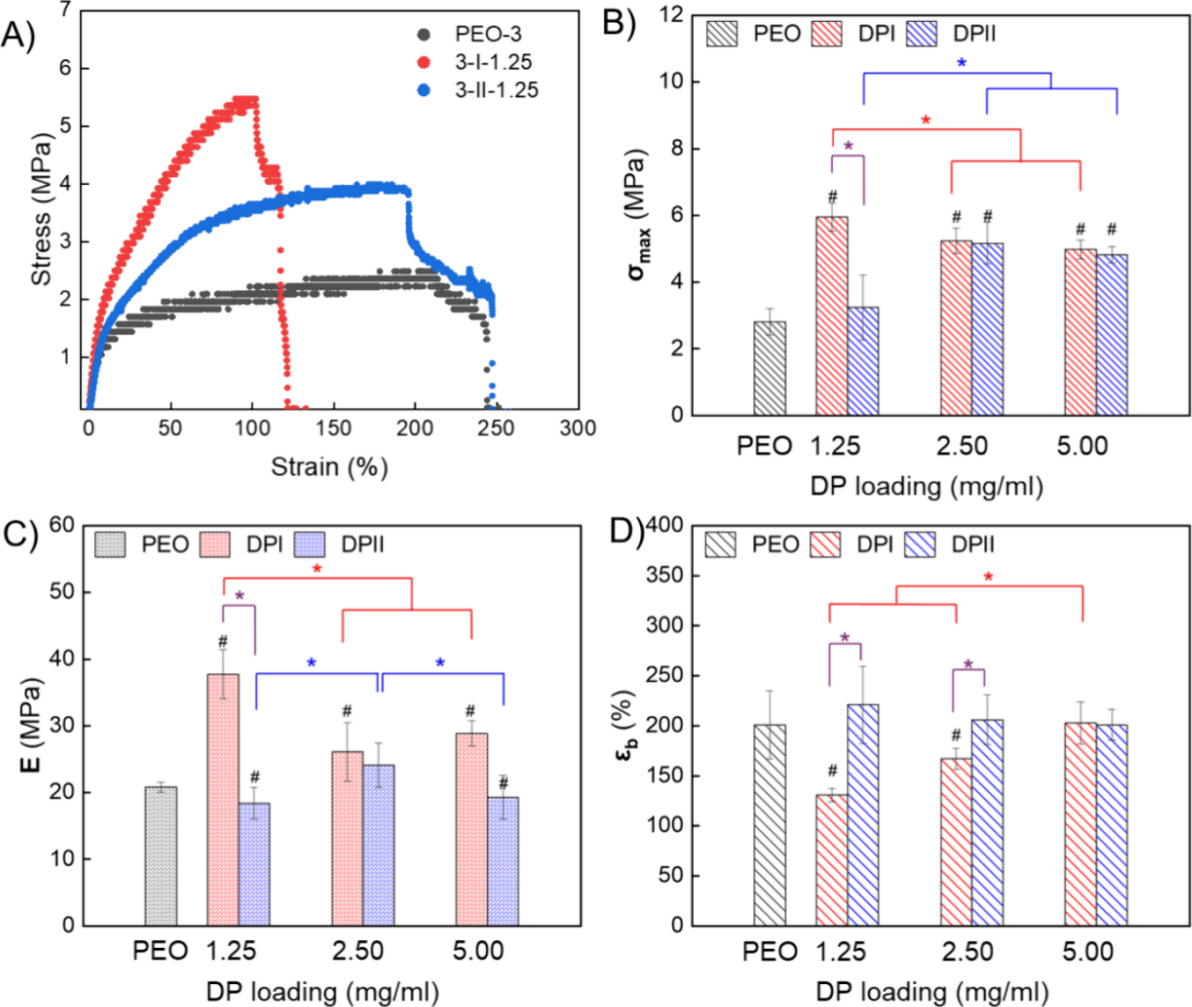
(A) Stress–strain curve of samples PEO-3, 3-I-1.25, and 3-II-1.25. (B) Comparison of σ_**max**_. (C) Comparison of *E*. (D) Comparison of ε_**b.**_ # means statistically different than PEO. Purple bracket: same loading level but different DP; red bracket: DP I at different loading; blue bracket: DP II at different loading; * indicates statistical significance at *p* < 0.05; samples collected from two independent runs with triplicates (*n* = 6). Overall, DP I significantly increases σ_**max**_ and ***E*** but reduces ε_**b**_ while DP II increases σ_**max**_ at high loading but does not affect ***E*** and ε_**b**._.

We previously reported nanomechanical evaluation of DP I fibrils and measured Youngs’ modulus (***E***) > 3 GPa.^41^ Using the designed collector (detailed in the Experimental section), electrospun mats were fabricated into uniform long strips for tensile testing. The tensile properties of the composite mats are shown in Figure 3 and summarized in Table 2. The tensile properties of the control PEO-3 mat (σ_**max**_ = 2.81 ± 0.39 MPa, ***E****=* 20.8 ± 0.8 MPa, elongation at break (ε_**b**_) = 201 ± 34%) align well with the values reported by Zhou et al. for PEO (σ_**max**_ = 2.05 ± 0.06 MPa, ***E****=* 15.2 ± 0.3 MPa, ε_**b**_ = 200 ± 12%).^50^ At all concentrations of DP I and DP II, the average modulus is higher than that of the neat PEO-3 mat (*p* < 0.05), and DP I generally provides a higher modulus than DP II. Notably, the lowest DP I loading level resulted in the highest ***E***, the lowest ε_**b**_, and the largest fiber diameter (sample 3-I-1.25, ***E*** = 37.6 ± 3.7 MPa, ε_**b**_ = 131 ± 7%). With increasing DP I concentration, ε_**b**_ increased while ***E*** decreased, but still remained higher than that of neat PEO. This negative correlation between peptide concentration and ***E*** is consistent with findings by Kaur et al, who observed a similar trend with their peptides that formed fibrous networks.^39^ The author hypothesized that the dispersion and orientation of peptide fibers in the polymer matrix affected the mechanical properties of the electrospun mat without providing a detailed explanation. Zhou et al. also reported this behavior for electrospun PEO/cellulose nanocrystal (CNC) composite mats, where increasing CNC loading led to higher ***E*** but lower **ε**_**b**_.^50^ Their morphology investigation showed that the rod-shaped CNCs were well-dispersed in the as-spun nanofibers and highly aligned along the nanofiber long-axis. In our system, we attribute the decrease in modulus as DP I concentration increases to the narrowing of the diameter of the fiber due to increased solution charge. Introducing DP II increased σ_**max**_ but did not significantly affect ***E*** or ε_**b**_. A similar pattern has been reported in PEO/ZnO nanoparticle composite fibers by Pittarate et al.^51^ In our system, we attribute the smaller effect of DP II to its globular rather than fibrillar morphology.

**Table 2:** Tensile Properties of Composite Mats

sample ID	σ_**max**_/MPa	***E***/MPa	ε_**b**_ (%)
PEO-3	2.81 ± 0.39	20.8 ± 0.8	201 ± 34
PEO (literature)	2.05 ± 0.06	15.2 ± 0.3	200 ± 12
3-I-1.25	5.95 ± 0.42	37.6 ± 3.7	131 ± 7
3-I-2.5	5.24 ± 0.37	26.0 ± 4.4	167 ± 11
3-I-5	4.98 ± 0.28	28.9 ± 1.9	203 ± 21
3-II-1.25	3.23 ± 0.97	18.4 ± 2.3	221 ± 39
3-II-2.5	5.16 ± 0.63	24.0 ± 3.2	206 ± 25
3-II-5	4.82 ± 0.25	19.3 ± 3.3	201 ± 16

### Blend Viscosity

**Figure 4:**
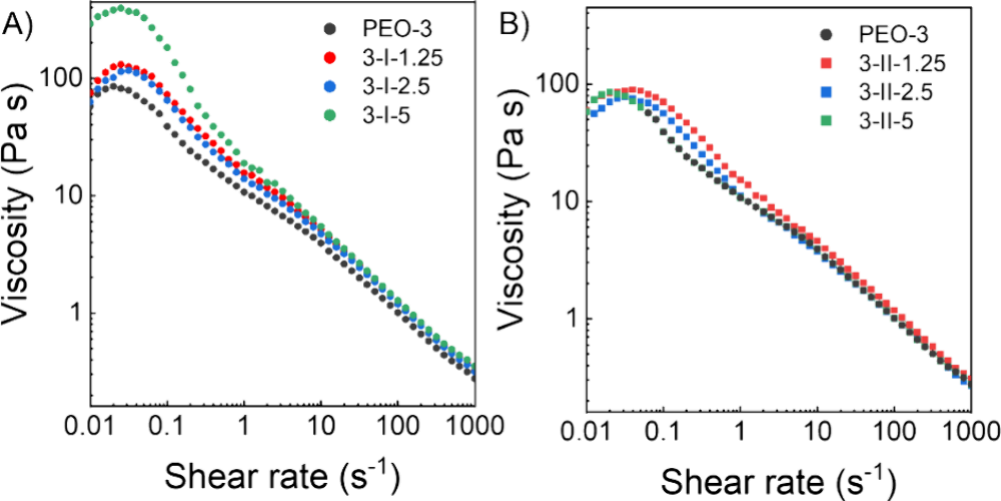
Solution viscosity plots of (A) PEO/DP I blends and (B) PEO/DP II blends. DP I increases the yield viscosity of the blend, and DP II does not have much effect on viscosity.

The apparent viscosity-shear rate relationship of the PEO/DP blends was determined to understand how peptide structure and assembled morphology influence the blend flow characteristics. Figure 4 illustrates that all blends and the control exhibit a yield viscosity at low shear rates, indicating the blend exceeds the entanglement concentration necessary for electrospinning. Adding DP I significantly increases the viscosity of the PEO/DP I blend, whereas DP II does not have a thickening effect. The viscosity-shear rate relationship and yield behavior development are influenced by the aspect ratio and loading level of the two DPs; AFM images demonstrate that DP I forms long fibril-like structures with moderate aggregation, while DP II forms well-dispersed nanometer-sized globules.^52,53^ Higher aspect ratio fillers, like those formed by DP I, percolate at lower loadings due to more efficient network formation, requiring fewer contacts to form a spanning cluster.^54^ We assume that above a critical concentration at low shear rates, DP I self-assembles to form a fibril network that results in a viscosity increase. This network is held together by physical bonds that are disrupted at higher shear rates. DP II may not reach its critical concentration at the levels tested and therefore, the viscosity profiles of PEO/DP II blends are highly overlaid with PEO-3. In electrospinning, the wall shear rate of non-Newtonian fluids is inversely proportional to the inner radius of the needle at a fixed volumetric flow rate.^50^ With an electrospinning flow rate of 0.25 mL/h using a 21G needle, the estimated shear rate is around 50/s. At high shear rates, the viscosity-shear rate relationship becomes independent of blend composition, explaining why the same electrospinning parameters can be used to fabricate nanofiber mats with varying blend compositions. The overall shear-thinning behavior can be attributed to the disentanglement of PEO chains during flow, the disruption of the moderate aggregation of DP I fibril-like bundles, and the alignment of the DP I fibrils in the direction of flow.^55^

### Peptide–Polymer Blends and Thermal Properties of Composite Mats

**Figure 5:**
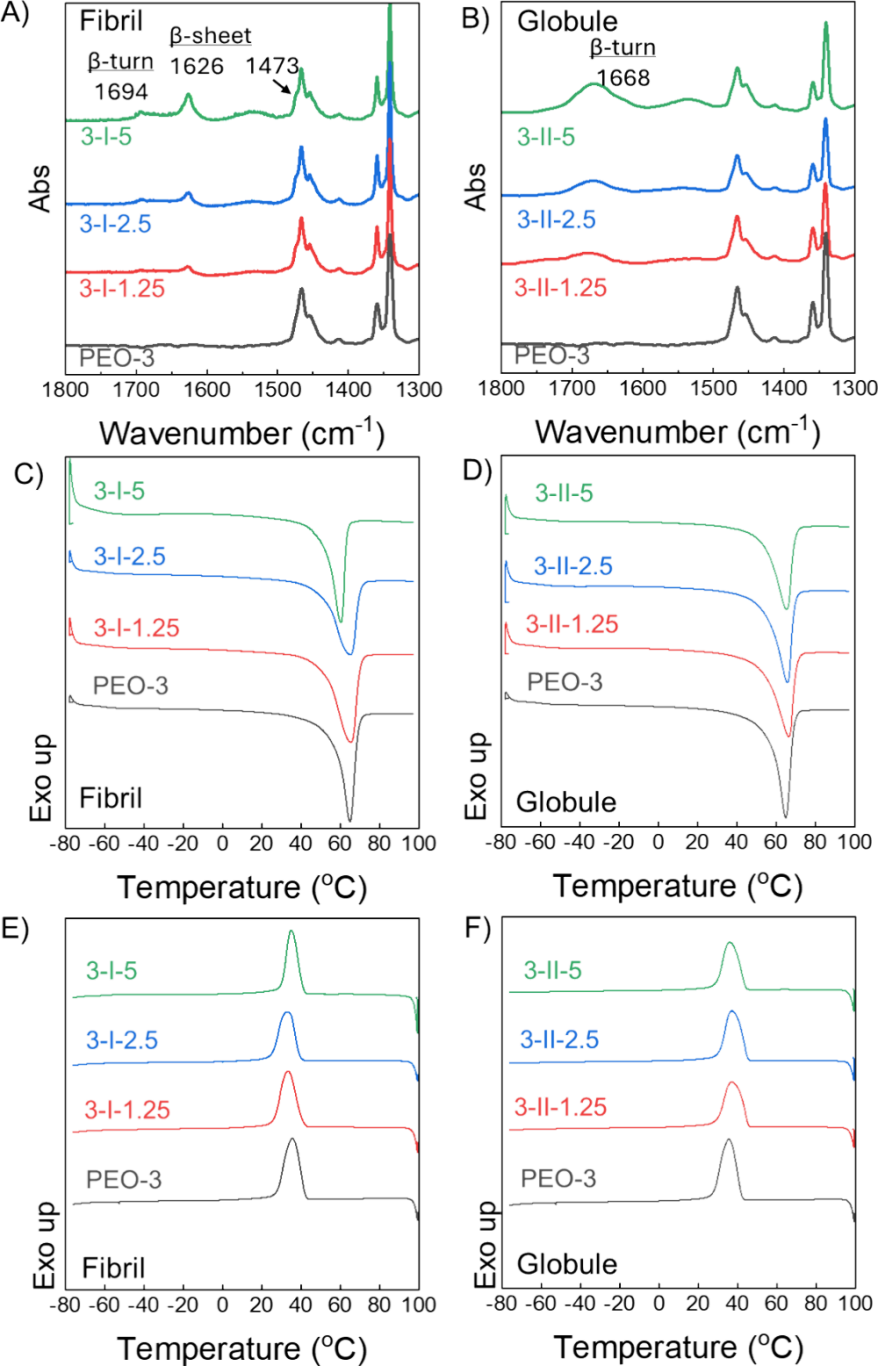
(A, B) ATR-FTIR of composite mats showing peptide secondary structures are preserved after electrospinning. (C, D) Melting curves from the second heating cycle, and (E, F) crystallization curves from the cooling cycle for composite mats compared to PEO-3. Incorporating DP does not dramatically affect the crystallization and melting temperatures but slightly broadens and reduces the melting peak.

To explain the observation of increased mechanical properties, the peptide incorporation and thermal properties of the composite mats were systematically examined. Figures 5A and B show ATR-FTIR characterization of electrospun mats to investigate the effects of processing on the DPs and potential interactions between the PEO and DPs. The solution FTIR spectrum of DP I alone showed bands at 1628 and 1681 cm^–1^ attributed to a mixture of parallel and antiparallel β-sheets (Figure 1C and Figure S3A).^45,46^ PEO/DP I composite mats show bands corresponding to parallel β-sheets at 1626 and a blue-shifted antiparallel band at 1694 cm^–1^ (+15 cm^–1^) for all blend proportions, with higher DP I ratios showing more intense antiparallel bands (Figure 5A and Figure S8).^56^ The observed blue shift is likely due to C = O exciton coupling in an antiparallel arrangement, suggesting the hydrogen bonding interactions of DP I are different in the hydrophobic PEO matrix than in the polar solution media. However, all PEO/DP II mats show a broad peak at 1668 cm^–1^, corresponding to β-turns with no β-sheet signatures. Peaks at 1468 and 1453 cm^–1^ are attributed to CH_2_ stretching in PEO.^57^ An additional small shoulder is apparent at 1473 cm^–1^ in the PEO/DP I electrospun mats, indicating hydrophobic interactions between DP I and PEO. Together, the data suggest that the peptide secondary structures are preserved after electrospinning with PEO.^46,56^

The thermal properties of composite mats were characterized using DSC to assess the impact of DP incorporation on the thermal phase transition behavior of PEO. Data for neat PEO and the PEO/DP blends are shown in Figure 5 C–F and summarized in Table 3. DSC traces of the neat DP I and DP II are shown in Figure S9. The electrospun sample PEO-3 exhibited a crystallization peak (*T*_c_) at 36 °C and a melting peak^58^ at 65 °C with 79% crystallinity, aligning with literature values.^50^ Only small differences in *T*_*c*_ are observed among the composite mats, with values ranging between 33 and 37 °C. Specifically, DP I composite mats show a slightly lower *T*_c_ than PEO-3, while DP II composite mats exhibit a slightly higher *T*_c_ than PEO-3. However, these differences are within one to two degrees. Measured melting temperature (*T*_m_) is unchanged for the composite mats in comparison to neat PEO, with the exception of the highest loading of DPI, where *T*_*m*_ was reduced by five degrees to 65 °C, suggesting increased PEO chain mobility. Significant decreases in the enthalpy of the melt transition and moderate broadening of the melting peaks are observed for all of the composite mats. The normalized degree of crystallinity is reduced from 79% to ∼60% for all composite mats, indicating an increase in the PEO amorphous phase. Neither DP I nor DP II exhibit phase transitions between −80 and 100 °C (Figure S9). The observed behavior for the PEO/DP mats is attributed to interactions between the peptides and PEO, and a similar suppressive effect on PEO crystallization was observed in blends of amphiphilic additives (such as fatty acids) with PEO.^59^

**Table 3:** Thermal Properties and Morphology of PEO/DP Composite Mat

sample ID	φ	enthalpy (J/g)	χ % (Normalized)	*T*_m_ (°C)	*T*_c_ (°C)
PEO-3	1.00	168	79	65	36
3-I-1.25	0.960	119	58	66	34
3-I-2.5	0.923	118	60	65	33
3-I-5	0.857	111	61	60	35
3-II-1.25	0.960	133	65	66	37
3-II-2.5	0.923	118	60	66	37
3-II-5	0.857	108	59	66	36

### Mesoscale Distribution of Peptides in PEO Matrix

**Figure 6:**
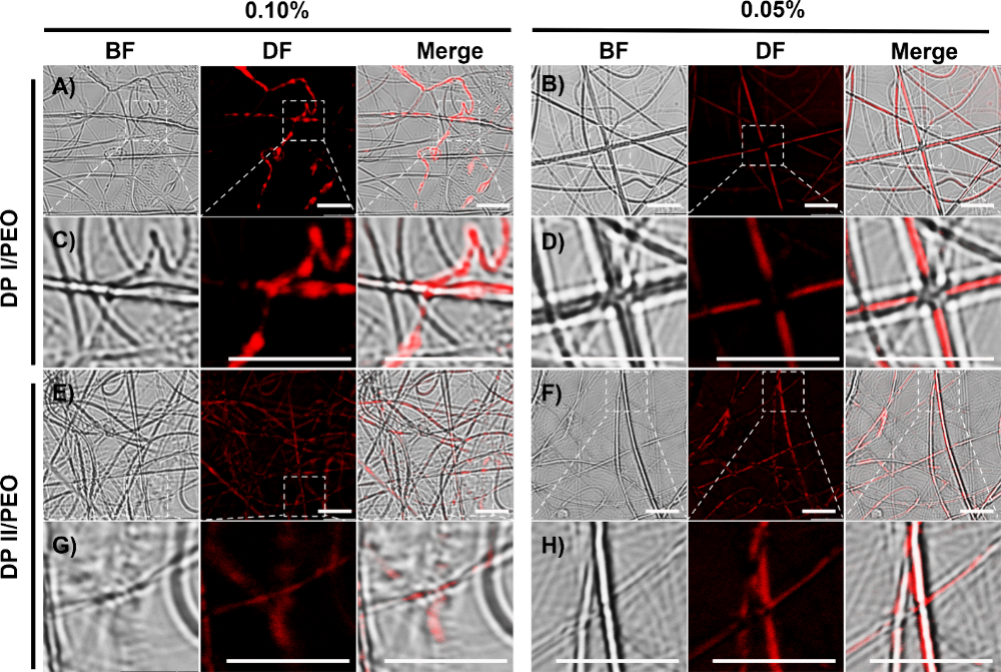
Morphology of PEO matrix electrospun with DPs: bright field (BF), dark field,^58^ and merged images under fluorescence confocal microscopy. (A, B) Blends of DP I with PEO matrix with 0.1% labeled (A) or 0.05% labeled peptide (B). (C, D) Zoomed in areas in panels (A) and (B), respectively. (E, F) Blends of DP II with PEO matrix with 0.1% labeled (A) or 0.05% labeled peptide (B). (G, H) Zoomed in areas in panels (E) and (F), respectively. All scale bars are 5 μm.

Incorporating DPs in the PEO mats leads to an overall lower degree of crystallinity, indicating that mechanical property enhancement is related to DP distribution. To further investigate this system, fluorescently labeled DP I and DP II were coincubated with unlabeled DPs at two different proportions, blended with PEO and electrospun onto a glass coverslip, and analyzed using fluorescence microscopy. Since the optimal loading level of the labeled peptide after extensive coincubation was unknown, two levels of fluorescently labeled DPs (0.1% and 0.05%) were screened, and fluorescence images are shown in Figure 6. Overall, both DPs showed good dispersion in the PEO matrix after electrospinning. It is important to note that the glass coverslip is less conductive than the metal collector used previously, so the deposition pattern and fiber morphology observed are expected to differ from those seen in the original SEMs. DP I and DP II have assembled dimensions much smaller than the diameter of nanofibers collected on the glass coverslip. Therefore, under 100× magnification, individual DP I fibrils and DP II globules cannot be distinguished; instead, a blurred fluorescence signal is observed in the PEO matrix. Based on this observation, we attribute the enhanced mechanical properties to the good dispersion of peptides within the PEO matrix, which enables efficient stress transfer, and the fibrillar DP I provides greater reinforcement than the globular DP II.

The combined FTIR, DSC, viscosity, and morphology studies indicate that decapeptide self-assembly and PEO/peptide interactions determine the tensile properties of the PEO/peptide mats. High molecular weight PEO is an amphiphilic nonionic polymer with good spinnability. DP I is considered a peptide amphiphile with polar ends and a hydrophobic central sequence. Subtle conformational differences observed via FTIR between the DP I in solution state and in the PEO/DP I mats suggest the presence of hydrophobic interactions between ethyl groups of the PEO backbone and DP I hydrophobic sections similar to those reported between hydrophobic patches of DP I assembled structures.^41^ The H-bonding interactions and hydrophobic interactions between the PEO and DP side groups can affect the crystallization behavior of PEO as observed in DSC.^60,61^ DP II shows amphiphilicity with random polar–apolar sequence distribution, and hydrophobic interactions with PEO likely aid in dispersion of this peptide. DP I self-assembles above a critical concentration to form fibrillar networks in PEO, observed as an increased yield viscosity at low shear rates. At high shear rates the physical network is disrupted and the shear thinning profile is identical to that of neat PEO. It is likely that at the high shear rates of electrospinning, DP I fibrils are oriented in the flow direction.^40,55^ DP II forms only spherical particles, which do not affect the viscosity profile. Fluorescence studies indicate that both peptides are well dispersed in the PEO matrix.

After electrospinning, well-dispersed DP I fibrils serve as high aspect ratio, stiff fillers, efficiently enhancing tensile properties of the composite mat. It could be hypothesized that DP I, a peptide amphiphile, might also serve as a plasticizer, as at high loading level (sample 3-I-5) the composite mat shows a reduced melting temperature compared to pure PEO nanofiber. However, this same sample shows increased modulus and maximum stress but equivalent elongation to neat PEO in tensile testing, behavior that is not consistent with a plasticizer. Therefore, the overall properties cannot be predicted solely by the plasticizing effect or the filler reinforcement effect. DP II does not reduce the PEO melting temperature. DP II behaves as a well-dispersed, hard spherical nanofiller, and at the highest level provides enhancement of the modulus and maximum stress without reducing elongation at break of the PEO nanofiber.

### Cytotoxicity Evaluation and Antioxidant Activity

**Figure 7:**
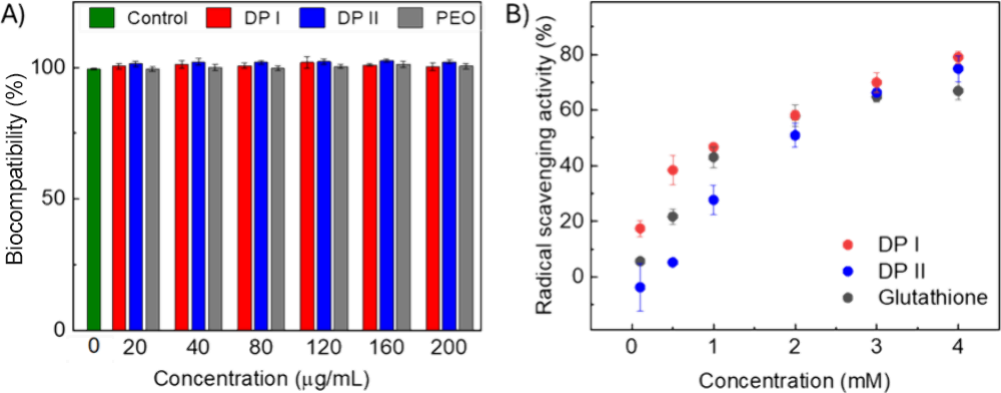
(A) Cytotoxicity evaluation of DPs. (B) Antioxidation evaluation of DPs. Both DPs show no induced toxicity to human embryonic kidney cells and good antioxidant activity.

Historically, amyloids are aggregates of proteins considered pathogenic and toxic to neurons, but many amyloid materials in nature have been shown to have beneficial features.^62^ To confirm the potential of these materials for biomedical applications, both DPs were evaluated for toxicity to human cells using a lactate dehydrogenase (LDH) assay, and results are shown in Figure 7A. Following 24 h of incubation with the cells, both DPs show no induced toxicity to human embryonic kidney cells (HEK 293) compared to control cells at all concentrations tested and no increase in toxicity compared to PEO. Thus, the presence of cationic segments did not impact the toxicity of the peptides, allowing applications, including wound dressings, to be considered for these highly biocompatible materials.

Free radicals accumulating at wound sites cause oxidative stress, which leads to lipid peroxidation, DNA damage, and enzyme inactivation, thereby impairing wound healing.^63^ The antioxidant activity of DPs was evaluated by comparing them to glutathione (the positive control) to explore their potential benefits as wound dressing materials. At low concentrations (0.1, 0.5, and 1 mM), DP I showed higher antioxidative activity than DP II at a statistically significant level, but no significant difference was observed at higher concentrations. DP I contains lysine and tryptophan, while DP II includes lysine, tryptophan, arginine, and methionine. Despite having more reductive pendant groups and theoretically stronger radical scavenging potential, DP II’s globular structures likely trap these pendant groups in the core, reducing their exposure on the surface and limiting their effectiveness. This comparison indicates that the higher-order assembled structure can significantly impact the peptide antioxidative performance beyond what might be predicted from their sequence alone.^64^ Such functionality would be useful for applications such as resorbable antioxidant dressings for wound treatment. Future studies of devices would, of course, be necessary for actual applications. Assessment of the assembled peptides is provided here as a proof of principle.

## Conclusions

In this research, two decapeptides with fibrillar (DP I) and globular (DP II) morphologies were blended with PEO ethanol/water solutions for electrospinning, and their morphological, mechanical, rheological, and thermal properties were systematically evaluated. The incorporation of DPs notably enhanced the mechanical properties of the composite mats, and such physical reinforcement seems to result from a uniform distribution of the DPs rather than changes in the crystallinity of the PEO matrix or nanofiber bulk alignment. The improved mechanical properties are impressive considering the thinner nanofibers in the composite mats. The lowered diameter in the amyloid containing fibers is attributed to increased conductivity of the electrospinning solution imparted by the zwitterionic nature of the peptides. Postprocessing analysis revealed that both decapeptides retained their secondary structures within the DP/PEO blends, indicating stability of DPs during electrospinning. Despite these similarities, DPs show subtle but distinct differences when blended with PEOs, which seem to correlate with their secondary structure and fibril-forming propensities. Low cytotoxicity and strong antioxidant activity of DPs underscore their potential for safe and effective use in the biomedical field. These findings highlight the intricate connections between peptide structural features, DP/PEO blend properties, and nanocomposite mat processing. This study also demonstrates the strong potential of using peptide–polymer blends for developing advanced biomaterials with applications in biotechnology, particularly in areas requiring enhanced mechanical properties and bioactivity.

## Experimental Section

### Materials

Wang polystyrene resin, Fmoc protected amino acids, and ethyl cyanoglyoxlate-2-oxime (Oxyma) were purchased from CEM peptides. Poly(ethylene oxide) (average *M*_v_ ∼ 1,000,000), 1,2-ethanedithiol (EDT), deuterated ethanol (C_2_D_5_OD) and deuterium oxide (D_2_O) were purchased from Sigma-Aldrich Corporation. Dichloromethane (DCM), diethyl ether, trifluoroacetic acid (TFA), dimethylformamide (DMF), ethanol, acetonitrile (MeCN), water (H_2_O, HPLC grade), Dulbecco’s modified eagle medium (DMEM), heat inactivated fetal bovine serum (FBS), penicillin-streptomycin, and CyQUANT lactate dehydrogenase (LDH) assay were all purchased from ThermoFisher. Diisopropylcarbodiimide (DIC) and triisopropylsilane were purchased from Acros Chemicals. 1,1-Diphenyl-2-picrylhydrazyl (DPPH) was purchased from Cayman Chemical Company. Sterile tissue treated 96-well plates were obtained from CellTreat. All chemicals were used without further purification.

### Peptide Synthesis and Purity Determination

Peptides were synthesized on a Liberty Blue 2.0 automated peptide synthesizer (CEM corporation) through standard 9-fluorenyl methoxycarbonyl (Fmoc)- based solid phase peptide synthesis as described in detail previously.^58,65,66^ Peptide synthesis was performed at 0.25 mmol scale using Fmoc-Asp(OtBu)-Wang Resin (LL) and Fmoc-Lys (Boc)-Wang Resin (LL) (0.3 mmol/g loading, 100–200 mesh, CEM corporation), for DP I (peptide sequence: KWVFLIFISD) and DP II (peptide sequence: KADERNMWAK), respectively. Deprotection of Fmoc protecting groups was carried out using 20 v/v% piperidine in DMF (90 °C microwave heating, 1 min deprotection, followed by 4 × DMF wash cycle). Each amino acid addition was carried out using Fmoc-protected amino acids (0.2 M), DIC (1M), and Oxyma (1 M) in DMF (90 °C microwave heating, 3 min coupling, followed by 4 × DMF wash cycle). After the final Fmoc deprotection, the resin beads were washed 3x using DCM. The peptide then underwent global deprotection and cleavage from the resin beads through gentle shaking in TFA/TIS/H_2_O/EDT (95:2.5:2.5:2.5) cleavage cocktail for 3 h at room temperature. Peptides were then precipitated in cold diethyl ether and collected via centrifugation (1500 rcf, 2 min, Ohaus Frontier 5706). The peptide pellet was then resuspended in diethyl ether and chilled for 4 h. It was recentrifuged, and the diethyl ether supernatant was decanted from the peptide pellet, which was allowed to air-dry. Crude peptides were purified by preparative reverse-phase high-performance liquid chromatography (HPLC) on a Prodigy system (CEM corporation) with a H_2_O/MeCN gradient (containing 0.1% TFA).^41^The mass and identity of the eluting fractions containing the desired DP peptides were confirmed using electrospray ionization-mass spectrometry^43^ on a Thermo Scientific Orbitrap Exploris 240. Peptide synthesis and purification were repeated at least three times to confirm reproducible yields and purities, as reported in our previous publication.^41^ Because of significant loss of peptide in the purification step, crude peptides were analyzed. The purity of the crude peptides was determined using liquid chromatography–mass spectrometry (LC-MS), shown in Figures S1 and S2. Labeling of decapeptides was carried out by incubating decapeptides (DP I and DP II) with 0.5x of fluorescent dye, HiLyte Fluor 647 succinimidyl ester *(AnaSpec Inc.)* for 12h at 4 °C. For the reactions, 0.1% and 0.05% fluorescent dye were used with the unlabeled decapeptides.

### Fourier Transform Infrared (FTIR) Spectroscopy

Two FTIR procedures were used in this study: one for liquid samples and one for solid samples. DP liquid samples were prepared at 2.5 mg/mL by dissolving the dry peptides in C_2_D_5_OD: D_2_O mixture (v/v = 3/1) and incubated at room temperature for 48 h. FTIR data on the DP solution was obtained at room temperature using an Agilent Cary 630 FTIR instrument equipped with a DialPath accessory. Recorded spectral range was from 1800–1300 cm^–1^ with an average of 1024 runs at 8 cm^–1^ resolution. For DP solution samples, the FTIR signal was processed by subtracting the background (i.e., C_2_D_5_OD: D_2_O mixture (v/v = 3/1) followed by baseline correction. Peak deconvolution was achieved by analyzing the first derivative using a smoothing function, and the wavenumber positions on the spectrum where the first-order derivative transitioned from positive to negative were detected and considered as potential peak candidates. The nonlinear curve fitting was applied using the Gaussian algorithm, with guidance from the first derivative analysis and this process was repeated until an acceptable R-squared value was achieved.^67^ ATR-FTIR spectra of composite mats were obtained using a Thermo Scientific Nicolet iS50 FT-IR in ATR mode with a diamond crystal with a recorded spectral range from 3800–800 cm^–1^ with an average of 128 scans at a resolution of 1 cm^–1^. For electrospun mats, the data was processed by subtracting the background (air), correcting the baseline, and normalizing with the min-max function. Full range spectra are shown in Figure S8.

### Atomic Force Microscopy (AFM)

AFM samples were prepared following a previously published procedure.^6^ Freshly cleaved mica substrates were first treated with 150 μL of APTES solution (500 μL of 3-aminopropyltriethoxysilane in 50 mL of 1 mM acetic acid) for 20 min. The APTES solution was decanted, and the surfaces were rinsed thrice with 150 μL H_2_O. The substrates were dried under a stream of N_2_ and stored in the desiccator for one h. Peptide solution (150 μL) was deposited onto the amine-treated mica substrates for 30 min to adsorb the peptide. The protein solution was then decanted, and the samples were rinsed three times with 150 μL H_2_O. The samples were dried under a stream of N_2_ and stored in the desiccator until imaging. All AFM experiments were performed using a Dimension Icon atomic force microscope (Bruker) in Standard Tapping Mode. AFM scanning was conducted using NanoScope 8.15r3sr9 software, and the images were analyzed using NanoScope Analysis 1.50 software. Imaging was performed using a sharp silicon nitride cantilever (RTESPA-300, nominal tip radius 8 nm; nominal resonance frequency of 300 kHz; nominal spring constant of 40 N/m) and a standard probe holder under ambient conditions with 512 × 512 data point resolution.

### PEO/Peptide Blend preparation

Table 4 summarizes the composition of each blend. The decapeptide was dissolved in ethanol/H_2_O mixture (v/v = 3/1) and incubated at room temperature for 48 h before mixing with PEO. The final electrospinning solution was kept at a total PEO concentration of 30 mg/mL (i.e., 3% w/v) in 3:1 ethanol/water with different peptide concentrations (1.25, 2.50, and 5.00 mg/mL). The 3 in the sample ID refers to 3 wt % of PEO in all formulations.

**Table 4:** Composition of PEO/DP Blends

sample ID	DP	DP conc. (mg/mL)	PEO: DP (wt. ratio)
PEO-3		0.00	100.0:0.0
3-I-1.25	DP I	1.25	96.0:4.0
3-I-2.5	DP I	2.50	92.3:7.7
3-I-5	DP I	5.00	85.5:14.2
3-II-1.25	DP II	1.25	96.0:4.0
3-II-2.5	DP II	2.50	92.3:7.7
3-II-5	DP II	5.00	85.5:14.2

### Solution Viscosity

Viscosity measurements were performed using a strain-controlled ARES rheometer with a cone-and-plate geometry (25 mm and 0.997 rad, or 50 mm and 0.499 rad). A steady rate sweep test was conducted at room temperature with a rate from 0.1 to 1000 s^–1^.

### Fabrication of PEO/Decapeptide Mats

Electrospun mats were fabricated using a Lab Mate 30 kV Electrospinning Machine (Spruce Science, CA, USA). Fibers were collected on a home-built aluminum drum collector with a collection width of 0.8 cm and a drum diameter of 7.5 cm, with a needle-collector distance of 22.5 cm and a rotation speed of 60 rpm, shown in Figure S3. The screening fabrication conditions were a 21G needle, 20 kV, and a flow rate of 0.25 mL/h.

### Scanning Electron Microscopy (SEM)

Analysis of the mat morphology was done using a Zeiss Sigma VP field-emission SEM with Thermo System 7 Energy Dispersive X-ray Spectroscopy (EDS) and Wavelength Dispersive X-ray Spectrometry (WDS) X-ray detectors (Thermo-Fisher Scientific, Waltham, MA). Electrospinning samples were carbon-coated using a Cressington Carbon Coater (coating pressure <0.01 mbar, coating duration ten seconds repeated ten times for each sample). The image was taken at 2K magnification. The fiber morphology was analyzed using ImageJ Analysis (ImageJ, National Institute of Health, MD), and the average fiber diameter was calculated by counting 100 fibers per image.

### Differential Scanning Calorimetry

The crystalline changes of electrospun fibers were monitored using a TA Instruments Discovery DSC 250 using aluminum hermetic pans. DSC thermograms were obtained using heat/cool/heat cycles from −80 to 100 °C with a heating ramp rate of 10 °C min^–1^ in all cycles. The electrospun mats were harvested and weighed without treatment. The DPs were first incubated in a 3:1 ethanol/H_2_O mixture at room temperature for 48 h for self-assembling, transferred to the DSC pan, and any residual solvent was removed via lyophilization. The degree of crystallinity (χ) was determined using eq 1:
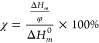
1where Δ*H*_*m*_^0^ = 213.7 J/g is the melting enthalpy when crystallinity of PEO is 100%, Δ*H*_*m*_ the melting enthalpy derived from the DSC curves, and φ is the weight fraction of PEO in the composite mat, (control: φ = 1; 1.25 mg/mL loading, φ = 0.96; 2.5 mg/mL loading, φ = 0.923; 5 mg/mL loading, φ = 0.857).^68^

### Solution Conductivity

The conductivity of the DP ethanol/water solution was measured using an Accumet XL 500 Dual Channel pH/mV/Ion/Conductivity meter. The measurement was conducted in single-point mode, with a cell constant of 1.000/cm, and each sample was measured three times. Calibration was performed using a conductivity standard of 147 μmhos/cm.

### Tensile Test

Tensile testing was performed using a Mark-10 EasyMESUR Test (Model F105) with a 25 N load cell. Electrospun mat samples were peeled from the collector, cut into strips with a length of 3.5 cm and width of 0.8 cm, and affixed to a paper frame using scotch tape, shown in Figure S4A. Samples were tested in at a pull-off rate of 13 mm/min. Mat thickness was estimated based on the mass normalization method and each sample’s thickness was calculated separately by weighing the broken strip after the tensile test.^69−71^ The stress was calculated using eqs 2 and 3:
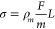
2
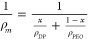
3where x is the mass fraction of DP, (1-x) is the mass fraction of PEO in the electrospun mats, ρ_*m*_ is the material bulk density, which is normalized based on eq 3, *m* is the specimen mass, *L* is the specimen initial length, *F* is the force, and σ is the stress. PEO density (ρ_*PEO*_) is 1.13 g/cm^3^, and the peptide density (ρ_*DP*_) is generally estimated as 1.35 g/cm^3^ as reported in literature.^72^ Tests were run in triplicate and samples were collected from two independent mat fabrications, n = 6.

### Fluorescence Microscopy and Sample Preparation

Fluorescence microscopic images of decapeptide fibers were obtained using Leica SP8 confocal microscope at 100× magnification. All images were processed using Affinity Designer. The labeled peptide (0.1%) was incubated with unlabeled peptide in a 3:1 ethanol/water mixture for 48 h prior to blending with PEO stock solution. The collector was changed to a flat aluminum plate with a glass cover (#1 micro cover glass VWR Scientific) placed on the top. After one hour of random fiber collection in the dark, the glass coverslip was carefully placed onto a plain microscope slide (Fisherbrand) to sandwich the fibers between the slide and coverslip. All glass substrates were cleaned with ethanol and wiped with lens paper prior to collection. The sample assembly was secured using instant dry topcoat (Sally Hansen) and stored in the dark prior to fluorescence imaging, shown in Figure S5.

### Cytotoxicity and Antioxidation Evaluation

Human embryonic kidney cells (HEK293) were grown to 90% confluence in tissue culture-treated polystyrene flasks in an incubator held at 37 °C with 5% CO_2_. Cells were grown in DMEM supplemented with 10% FBS and 1% penicillin-streptomycin. HEK293 cells were seeded in a 96-well plate (1 × 105 cells/mL, 100 μL volume per well) and were incubated at 37 °C and 5% CO_2_ overnight to allow cells to adhere. DP I, DP II, and PEO were dissolved in supplemented DMEM at 1 mg/mL to create a stock solution, followed by serial dilutions with DMEM to obtain the final treatment concentration with cells. Each concentration was assessed in triplicate. Nuclease-free water was used as a spontaneous LDH control, and Triton X-100 was used as a positive control for 100% cytotoxicity (i.e., complete LDH release). Plates were incubated for 24 h at 37 °C and 5% CO_2_ before collecting media to assess LDH release with the CyQUANT LDH assay following the manufacturers protocols.

DPPH solution (100 uM, in ethanol/water (V/V = 3:1)) was freshly prepared before use. Glutathione, a known antioxidant, was selected as the positive control. The DPPH radical-scavenging activity was determined using the method of Santos et al.^73^ DPPH solution (50 uL) was mixed with 50 uL analyte solutions (100, 500,1000, 2000, 3000, 4000 uM). The mixture was kept in a dark environment at room temperature for 30 min. The normalized DPPH intensity was calculated using eq 4:
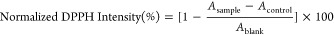
4where A_sample_ is the absorbance value of the 50 uL sample mixed with 50 uL DPPH solution, A_control_ is the absorbance value of 50 uL sample solution mixed with 50 uL ethanol/water solution, and A_blank_ is the absorbance value of 50 uL DPPH solution mixed with 50 uL ethanol/water solution alone.^74^ Lower normalized intensity means more DPPH radicals are quenched, indicating stronger antioxidant activity.

Statistical significance of the cytotoxicity data and antioxidant activity at each concentration was determined using one-way analysis of variance (ANOVA), and *p* < 0.05 was considered the significance level for statistical analysis.

## Supporting Information Available

(Figure S1) Chemical structure and molecular weight of DPI, LC-MS trace of crude DPI, corresponding crude ESI-mass spectra, LC-MS trace of pure DPI, and corresponding pure ESI-mass spectra; (Figure S2) chemical structure and molecular weight of DPII, LC-MS trace of crude DPII, corresponding crude ESI-mass spectra, LC-MS trace of pure DPII, and corresponding pure ESI-mass spectra; (Figure S3) FTIR spectra of DP I and DP II; (Figure S4) electrospinning setup; the collecting station can move horizontally while the collector rotates, and picture of the home-built collector; (Figure S5) additional SEM images of electrospun mats, 3-I-1.25, 3-I-2.5, 3-I-5, 3-II-1.25, 3-II-2.5, and 3-II-5; (Figure S6) paper frame used to prepare tensile bars and snapshot of the tensile test; (Figure S7) aluminum/glass cover as collector and sandwiched sample for imaging; (Figure S8) ATR-FTIR of PEO/DP I and PEO/DP II composite mats from 3800 to 700 wavenumber; (Figure S9) DSC traces of self-assembled DP I and DP 2; (Figure S10) AFM height image of DP I fibril with a scale bar of 400 nm; and (Table S1) conductivity of DP solutions (PDF)

## References

[ref1] Rambaran R. N.; Serpell L. C. Amyloid fibrils. Prion 2008, 2 (3), 112–117. 10.4161/pri.2.3.7488. 19158505 PMC2634529

[ref2] Balistreri A.; Goetzler E.; Chapman M. Functional Amyloids Are the Rule Rather Than the Exception in Cellular Biology. Microorganisms 2020, 8 (12), 1951 10.3390/microorganisms8121951. 33316961 PMC7764130

[ref3] Otzen D.; Riek R. Functional Amyloids. Cold Spring Harb. Perspect. Biol. 2019, 11 (12), a033860 10.1101/cshperspect.a033860. 31088827 PMC6886451

[ref4] Schleeger M.; vandenAkker C. C.; Deckert-Gaudig T.; Deckert V.; Velikov K. P.; Koenderink G.; Bonn M. Amyloids: From molecular structure to mechanical properties. Polymer 2013, 54 (10), 2473–2488. 10.1016/j.polymer.2013.02.029.

[ref5] Prosswimmer T.; Heng A.; Daggett V. Mechanistic insights into the role of amyloid-β in innate immunity. Sci. Rep. 2024, 14 (1), 5376 10.1038/s41598-024-55423-9. 38438446 PMC10912764

[ref6] Saha J.; Ford B. J.; Wang X.; Boyd S.; Morgan S. E.; Rangachari V. Sugar distributions on gangliosides guide the formation and stability of amyloid-β oligomers. Biophys. Chem. 2023, 300, 107073 10.1016/j.bpc.2023.107073. 37413816 PMC10529042

[ref7] Sabaté R.; Ventura S. Cross-β-Sheet Supersecondary Structure in Amyloid Folds: Techniques for Detection and Characterization. In Protein Supersecondary Structures; Methods in Molecular Biology; Kister A. E., Ed. Humana Press: Totowa, NJ, 2013; Vol. 932, pp 237–257. 10.1007/978-1-62703-065-6_15 22987357

[ref8] So̷nderby T. V.; Najarzadeh Z.; Otzen D. E. Functional Bacterial Amyloids: Understanding Fibrillation, Regulating Biofilm Fibril Formation and Organizing Surface Assemblies. Molecules 2022, 27 (13), 4080 10.3390/molecules27134080. 35807329 PMC9268375

[ref9] Peña-Díaz S.; Olsen W. P.; Wang H.; Otzen D. E. Functional Amyloids: The Biomaterials of Tomorrow?. Adv. Mater. 2024, 36 (18), 2312823 10.1002/adma.202312823. 38308110

[ref10] Rasmussen H. Ø.; Kumar A.; Shin B.; Stylianou F.; Sewell L.; Xu Y.; Otzen D. E.; Pedersen J. S.; Matthews S. J. FapA is an Intrinsically Disordered Chaperone for Pseudomonas Functional Amyloid FapC. J. Mol. Biol. 2023, 435 (2), 167878 10.1016/j.jmb.2022.167878. 36368411

[ref11] Chen D.; Liu X.; Chen Y.; Lin H. Amyloid peptides with antimicrobial and/or microbial agglutination activity. Appl. Microbiol. Biotechnol. 2022, 106 (23), 7711–7720. 10.1007/s00253-022-12246-w. 36322251 PMC9628408

[ref12] Gosztyla M. L.; Brothers H. M.; Robinson S. R. Alzheimer’s Amyloid-β is an Antimicrobial Peptide: A Review of the Evidence. J. Alzheimer’s Dis. 2018, 62 (4), 1495–1506. 10.3233/JAD-171133. 29504537

[ref13] Sciacca M. F. M.; La Rosa C.; Milardi D. Amyloid-Mediated Mechanisms of Membrane Disruption. Biophysica 2021, 1 (2), 137–156. 10.3390/biophysica1020011.

[ref14] Kikuchi T.; Mizunoe Y.; Takade A.; Naito S.; Yoshida S.-i. Curli Fibers Are Required for Development of Biofilm Architecture in Escherichia coli K-12 and Enhance Bacterial Adherence to Human Uroepithelial Cells. Microbiol. Immunol. 2005, 49 (9), 875–884. 10.1111/j.1348-0421.2005.tb03678.x. 16172544

[ref15] Van Houdt R.; Michiels C. W. Role of bacterial cell surface structures in Escherichia coli biofilm formation. Res. Microbiol. 2005, 156 (5), 626–633. 10.1016/j.resmic.2005.02.005. 15950122

[ref16] Wang A.; Keten S. Adhesive behavior and detachment mechanisms of bacterial amyloid nanofibers. npj Comput. Mater. 2019, 5 (1), 29 10.1038/s41524-019-0154-7.

[ref17] Shanmugam N.; Baker M. O. D. G.; Ball S. R.; Steain M.; Pham C. L. L.; Sunde M. Microbial functional amyloids serve diverse purposes for structure, adhesion and defence. Biophys. Rev. 2019, 11 (3), 287–302. 10.1007/s12551-019-00526-1. 31049855 PMC6557962

[ref18] Liu X.; Liang C.; Zhang X.; Li J.; Huang J.; Zeng L.; Ye Z.; Hu B.; Wu W. Amyloid fibril aggregation: An insight into the underwater adhesion of barnacle cement. Biochem. Biophys. Res. Commun. 2017, 493 (1), 654–659. 10.1016/j.bbrc.2017.08.136. 28865959

[ref19] Zhong C.; Gurry T.; Cheng A. A.; Downey J.; Deng Z.; Stultz C. M.; Lu T. K. Strong underwater adhesives made by self-assembling multi-protein nanofibres. Nat. Nanotechnol. 2014, 9 (10), 858–866. 10.1038/nnano.2014.199. 25240674 PMC4191913

[ref20] Knowles T. P.; Fitzpatrick A. W.; Meehan S.; Mott H. R.; Vendruscolo M.; Dobson C. M.; Welland M. E. Role of Intermolecular Forces in Defining Material Properties of Protein Nanofibrils. Science 2007, 318 (5858), 1900–1903. 10.1126/science.1150057. 18096801

[ref21] Qi X.; Lv X.; Pan W.; Shen M.; Chen Y.; Yu Q.; Xie J. Antioxidant amyloid fibril derived from rice protein hydrolysate as stabilizer towards preparing high-stable emulsion. Food Chem. 2024, 460, 140745 10.1016/j.foodchem.2024.140745. 39126945

[ref22] Tong X.; Cao J.; Tian T.; Lyu B.; Miao L.; Lian Z.; Cui W.; Liu S.; Wang H.; Jiang L. Changes in structure, rheological property and antioxidant activity of soy protein isolate fibrils by ultrasound pretreatment and EGCG. Food Hydrocolloids 2022, 122, 107084 10.1016/j.foodhyd.2021.107084.

[ref23] Mohammadian M.; Madadlou A. Characterization of fibrillated antioxidant whey protein hydrolysate and comparison with fibrillated protein solution. Food Hydrocolloids 2016, 52, 221–230. 10.1016/j.foodhyd.2015.06.022.

[ref24] Zhao Y.; Wang C.; Lu W.; Sun C.; Zhu X.; Fang Y. Evolution of physicochemical and antioxidant properties of whey protein isolate during fibrillization process. Food Chem. 2021, 357, 129751 10.1016/j.foodchem.2021.129751. 33872866

[ref25] Wang W.; Azizyan R. A.; Garro A.; Kajava A. V.; Ventura S. Multifunctional Amyloid Oligomeric Nanoparticles for Specific Cell Targeting and Drug Delivery. Biomacromolecules 2020, 21 (10), 4302–4312. 10.1021/acs.biomac.0c01103. 32885960

[ref26] Diaz C.; Missirlis D. Amyloid-Based Albumin Hydrogels. Adv. Healthcare Mater. 2023, 12 (7), 2201748 10.1002/adhm.202201748. PMC11469233 36469813

[ref27] Das S.; Jacob R. S.; Patel K.; Singh N.; Maji S. K. Amyloid Fibrils: Versatile Biomaterials for Cell Adhesion and Tissue Engineering Applications. Biomacromolecules 2018, 19 (6), 1826–1839. 10.1021/acs.biomac.8b00279. 29701992

[ref28] Peydayesh M.; Suter M. K.; Bolisetty S.; Boulos S.; Handschin S.; Nyström L.; Mezzenga R. Amyloid Fibrils Aerogel for Sustainable Removal of Organic Contaminants from Water. Adv. Mater. 2020, 32 (12), 1907932 10.1002/adma.201907932. 32026524

[ref29] Bolisetty S.; Reinhold N.; Zeder C.; Orozco M. N.; Mezzenga R. Efficient purification of arsenic-contaminated water using amyloid–carbon hybrid membranes. Chem. Commun. 2017, 53 (42), 5714–5717. 10.1039/C7CC00406K. 28487912

[ref30] Jia X.; Peydayesh M.; Huang Q.; Mezzenga R. Amyloid Fibril Templated MOF Aerogels for Water Purification. Small 2022, 18 (4), 2105502 10.1002/smll.202105502. 34816591

[ref31] Men D.; Guo Y.-C.; Zhang Z.-P.; Wei H.-p.; Zhou Y.-F.; Cui Z.-Q.; Liang X.-S.; Li K.; Leng Y.; You X.-Y.; Zhang X.-E. Seeding-Induced Self-assembling Protein Nanowires Dramatically Increase the Sensitivity of Immunoassays. Nano Lett. 2009, 9 (6), 2246–2250. 10.1021/nl9003464. 19402649

[ref32] Saldanha D. J.; Abdali Z.; Modafferi D.; Janfeshan B.; Dorval Courchesne N.-M. Fabrication of fluorescent pH-responsive protein–textile composites. Sci. Rep. 2020, 10 (1), 13052 10.1038/s41598-020-70079-x. 32747732 PMC7400762

[ref33] Li T.; Kambanis J.; Sorenson T. L.; Sunde M.; Shen Y. From Fundamental Amyloid Protein Self-Assembly to Development of Bioplastics. Biomacromolecules 2024, 25 (1), 5–23. 10.1021/acs.biomac.3c01129. 38147506 PMC10777412

[ref34] Peydayesh M.; Bagnani M.; Mezzenga R. Sustainable Bioplastics from Amyloid Fibril-Biodegradable Polymer Blends. ACS Sustainable Chem. Eng. 2021, 9 (35), 11916–11926. 10.1021/acssuschemeng.1c03937.

[ref35] Bagnani M.; Ehrengruber S.; Soon W. L.; Peydayesh M.; Miserez A.; Mezzenga R. Rapeseed Cake Valorization into Bioplastics Based on Protein Amyloid Fibrils. Adv. Mater. Technol. 2023, 8 (3), 2200932 10.1002/admt.202200932.

[ref36] Bucci R.; Georgilis E.; Bittner A. M.; Gelmi M. L.; Clerici F. Peptide-Based Electrospun Fibers: Current Status and Emerging Developments. Nanomaterials 2021, 11, 1262 10.3390/nano11051262. 34065019 PMC8151459

[ref37] Brun P.; Ghezzo F.; Roso M.; Danesin R.; Palu G.; Bagno A.; Modesti M.; Castagliuolo I.; Dettin M. Electrospun scaffolds of self-assembling peptides with poly(ethylene oxide) for bone tissue engineering. Acta Biomaterialia 2011, 7, 2526–2532. 10.1016/j.actbio.2011.02.025. 21345384

[ref38] Rubin D. J.; Nia H. T.; Desire T.; Nguyen P. Q.; Gevelber M.; Ortiz C.; Joshi N. S. Mechanical Reinforcement of Polymeric Fibers through Peptide Nanotube Incorporation. Biomacromolecules 2013, 14 (10), 3370–3375. 10.1021/bm4008293. 24070499

[ref39] Kaur G.; Kumari S.; Saha P.; Ali R.; Patil S.; Ganesh S.; Verma S. Selective Cell Adhesion on Peptide–Polymer Electrospun Fiber Mats. ACS Omega 2019, 4 (2), 4376–4383. 10.1021/acsomega.8b03494.

[ref40] Kim K.; Kloxin C. J.; Saven J. G.; Pochan D. J. Nanofibers Produced by Electrospinning of Ultrarigid Polymer Rods Made from Designed Peptide Bundlemers. ACS Appl. Mater. Interfaces 2021, 13, 26339–26351. 10.1021/acsami.1c04027. 34029045

[ref41] Abernathy H. G.; Saha J.; Kemp L. K.; Wadhwani P.; Clemons T. D.; Morgan S. E.; Rangachari V. De novo amyloid peptides with subtle sequence variations differ in their self-assembly and nanomechanical properties. Soft Matter 2023, 19 (27), 5150–5159. 10.1039/D3SM00604B. 37386911

[ref42] Gizaw M.; Thompson J.; Faglie A.; Lee S.-Y.; Neuenschwander P.; Chou S.-F. Electrospun Fibers as a Dressing Material for Drug and Biological Agent Delivery in Wound Healing Applications. Bioengineering 2018, 5 (1), 9 10.3390/bioengineering5010009. 29382065 PMC5874875

[ref43] Yadav S. S.; Padhy P. K.; Singh A. K.; Sharma S.; Tanu; Fatima S.; Sinha A.; Tariq R.; Varsha; Sharma S. K.; Priya S. Advancements in amyloid-based biological materials for healthcare, environmental and sensing applications. Mater. Adv. 2024, 5 (10), 4078–4090. 10.1039/D3MA00969F.

[ref44] Sethuram L.; Thomas J. Therapeutic applications of electrospun nanofibers impregnated with various biological macromolecules for effective wound healing strategy – A review. Biomed. Pharmacother. 2023, 157, 113996 10.1016/j.biopha.2022.113996. 36399827

[ref45] Kong J.; Yu S. Fourier Transform Infrared Spectroscopic Analysis of Protein Seconday Structures. Acta Biochim. Biophys. Sin. 2007, 39 (8), 549–550. 10.1111/j.1745-7270.2007.00320.x. 17687489

[ref46] Jackson M.; Mantsch H. H. The Use and Misuse of FTIR Spectroscopy in the Determination of Protein Structure. Crit. Rev. Biochem. Mol. Biol. 1995, 30 (2), 95–120. 10.3109/10409239509085140. 7656562

[ref47] Topuz F.; Abdulhamid M. A.; Holtzl T.; Szekely G. Nanofiber engineering of microporous polyimides through electrospinning: Influence of electrospinning parameters and salt addition. Mater. Des. 2021, 198, 109280 10.1016/j.matdes.2020.109280.

[ref48] Qin X.-H.; Yang E.-L.; Li N.; Wang S.-Y. Effect of different salts on electrospinning of polyacrylonitrile (PAN) polymer solution. J. Appl. Polym. Sci. 2007, 103 (6), 3865–3870. 10.1002/app.25498.

[ref49] Zong X.; Kim K.; Fang D.; Ran S.; Hsiao B. S.; Chu B. Structure and process relationship of electrospun bioabsorbable nanofiber membranes. Polymer 2002, 43 (16), 4403–4412. 10.1016/S0032-3861(02)00275-6.

[ref50] Zhou C.; Chu R.; Wu R.; Wu Q. Electrospun Polyethylene Oxide/Cellulose Nanocrystal Composite Nanofibrous Mats with Homogeneous and Heterogeneous Microstructures. Biomacromolecules 2011, 12 (7), 2617–2625. 10.1021/bm200401p. 21574638

[ref51] Pittarate C.; Yoovidhya T.; Srichumpuang W.; Intasanta N.; Wongsasulak S. Effects of poly(ethylene oxide) and ZnO nanoparticles on the morphology, tensile and thermal properties of cellulose acetate nanocomposite fibrous film. Polym. J. 2011, 43 (12), 978–986. 10.1038/pj.2011.97.

[ref52] Samal S.; Kim S.; Kim H. Effects of Filler Size and Distribution on Viscous Behavior of Glass Composites. J. Am. Ceram. Soc. 2012, 95 (5), 1595–1603. 10.1111/j.1551-2916.2012.05181.x.

[ref53] Kataoka T.; Kitano T.; Sasahara M.; Nishijima K. Viscosity of particle filled polymer melts. Rheol. Acta 1978, 17 (2), 149–155. 10.1007/BF01517705.

[ref54] Mutiso R. M.; Winey K. I. Electrical Conductivity of Polymer Nanocomposites. In Polymer Science: A Comprehensive Reference, Matyjaszewski K.; Möller M., Eds. Elsevier: Amsterdam, 2012; Vol. 7, pp 327–344.

[ref55] Chen D.; Narayanan N.; Federici E.; Yang Z.; Zuo X.; Gao J.; Fang F.; Deng M.; Campanella O. H.; Jones O. G. Electrospinning Induced Orientation of Protein Fibrils. Biomacromolecules 2020, 21 (7), 2772–2785. 10.1021/acs.biomac.0c00500. 32463660

[ref56] Sarroukh R.; Goormaghtigh E.; Ruysschaert J.-M.; Raussens V. ATR-FTIR: A “rejuvenated” tool to investigate amyloid proteins. Biochim. Biophys. Acta, Biomemb. 2013, 1828 (10), 2328–2338. 10.1016/j.bbamem.2013.04.012. 23746423

[ref57] Sim L. H.; Gan S. N.; Chan C. H.; Yahya R. ATR-FTIR studies on ion interaction of lithium perchlorate in polyacrylate/poly(ethylene oxide) blends. Spectrochim. Acta A Mol. Biomol. Spectrosc. 2010, 76 (3), 287–292. 10.1016/j.saa.2009.09.031. 20444642

[ref58] Mondal M.; Jankoski P. E.; Lee L. D.; Dinakarapandian D. M.; Chiu T.-Y.; Swetman W. S.; Wu H.; Paravastu A. K.; Clemons T. D.; Rangachari V. Reversible Disulfide Bond Cross-Links as Tunable Levers of Phase Separation in Designer Biomolecular Condensates. J. Am. Chem. Soc. 2024, 146 (36), 25299–25311. 10.1021/jacs.4c09557. 39196681 PMC11403603

[ref59] Pielichowska K.; Głowinkowski S.; Lekki J.; Biniaś D.; Pielichowski K.; Jenczyk J. PEO/fatty acid blends for thermal energy storage materials. Structural/morphological features and hydrogen interactions. Eur. Polym. J. 2008, 44 (10), 3344–3360. 10.1016/j.eurpolymj.2008.07.047.

[ref60] Spitalsky Z.; Tasis D.; Papagelis K.; Galiotis C. Carbon nanotube–polymer composites: Chemistry, processing, mechanical and electrical properties. Prog. Polym. Sci. 2010, 35 (3), 357–401. 10.1016/j.progpolymsci.2009.09.003.

[ref61] Rahmat M.; Hubert P. Carbon nanotube–polymer interactions in nanocomposites: A review. Compos. Sci. Technol. 2011, 72 (1), 72–84. 10.1016/j.compscitech.2011.10.002.

[ref62] Yakupova E. I.; Bobyleva L. G.; Shumeyko S. A.; Vikhlyantsev I. M.; Bobylev A. G. Amyloids: The History of Toxicity and Functionality. Biology 2021, 10 (5), 394 10.3390/biology10050394. 34062910 PMC8147320

[ref63] Zhao Y.; Li X.; Sun N.; Mao Y.; Ma T.; Liu X.; Cheng T.; Shao X.; Zhang H.; Huang X.; Li J.; Huang N.; Wang H. Injectable Double Crosslinked Hydrogel-Polypropylene Composite Mesh for Repairing Full-Thickness Abdominal Wall Defects. Adv. Healthcare Mater. 2024, 13 (15), 2304489 10.1002/adhm.202304489. 38433421

[ref64] Zou T.-B.; He T.-P.; Li H.-B.; Tang H.-W.; Xia E.-Q. The Structure-Activity Relationship of the Antioxidant Peptides from Natural Proteins. Molecules 2016, 21 (1), 72 10.3390/molecules21010072. 26771594 PMC6273900

[ref65] Clemons T. D.; Egner S. A.; Grzybek J.; Roan J. J.; Sai H.; Yang Y.; Syrgiannis Z.; Sun H.; Palmer L. C.; Gianneschi N. C.; Stupp S. I. Hybrid Bonding Bottlebrush Polymers Grafted from a Supramolecular Polymer Backbone. J. Am. Chem. Soc. 2024, 146 (23), 16085–16096. 10.1021/jacs.4c03320. 38831660

[ref66] Edelbrock A. N.; Clemons T. D.; Chin S. M.; Roan J. J. W.; Bruckner E. P.; Álvarez Z.; Edelbrock J. F.; Wek K. S.; Stupp S. I. Superstructured Biomaterials Formed by Exchange Dynamics and Host–Guest Interactions in Supramolecular Polymers. Adv. Sci. 2021, 8 (8), 2004042 10.1002/advs.202004042. PMC8061421 33898187

[ref67] Miller L. M.; Bourassa M. W.; Smith R. J. FTIR spectroscopic imaging of protein aggregation in living cells. Biochim. Biophys. Acta, Biomemb. 2013, 1828 (10), 2339–2346. 10.1016/j.bbamem.2013.01.014. PMC3722250 23357359

[ref68] Money B. K.; Swenson J. Dynamics of Poly(ethylene oxide) around Its Melting Temperature. Macromolecules 2013, 46 (17), 6949–6954. 10.1021/ma4003598.

[ref69] Maccaferri E.; Cocchi D.; Mazzocchetti L.; Benelli T.; Brugo T. M.; Giorgini L.; Zucchelli A. How Nanofibers Carry the Load: Toward a Universal and Reliable Approach for Tensile Testing of Polymeric Nanofibrous Membranes. Macromol. Mater. Eng. 2021, 306 (7), 2100183 10.1002/mame.202100183.

[ref70] Munawar M. A.; Schubert D. W. Highly Oriented Electrospun Conductive Nanofibers of Biodegradable Polymers-Revealing the Electrical Percolation Thresholds. ACS Appl. Polym. Mater. 2021, 3 (6), 2889–2901. 10.1021/acsapm.0c01332.

[ref71] Maccaferri E.; Ortolani J.; Mazzocchetti L.; Benelli T.; Brugo T. M.; Zucchelli A.; Giorgini L. New Application Field of Polyethylene Oxide: PEO Nanofibers as Epoxy Toughener for Effective CFRP Delamination Resistance Improvement. ACS Omega 2022, 7 (27), 23189–23200. 10.1021/acsomega.2c01189. 35847344 PMC9281329

[ref72] Fischer H.; Polikarpov I.; Craievich A. F. Average protein density is a molecular-weight-dependent function. Protein Sci. 2004, 13 (10), 2825–2828. 10.1110/ps.04688204. 15388866 PMC2286542

[ref73] Santos J. S.; Alvarenga Brizola V. R.; Granato D. High-throughput assay comparison and standardization for metal chelating capacity screening: A proposal and application. Food Chem. 2017, 214, 515–522. 10.1016/j.foodchem.2016.07.091. 27507505

[ref74] Yang L.; Xing Y.; Chen R.; Ni H.; Li H.-H. Isolation and identification of antioxidative peptides from crocodile meat hydrolysates using silica gel chromatography. Sci. Rep. 2022, 12 (1), 13223 10.1038/s41598-022-16009-5. 35918356 PMC9345901

