# Supplementary material for: DeNovo Amyloid Peptide–Polymer Blends with Enhanced Mechanical and Biological Properties
Source: ACS Appl Polym Mater. 2025 Mar 12;7(6):3739–51. doi: 10.1021/acsapm.4c04020 (PMC11959523; doi:10.1021/acsapm.4c04020)
Supplement: Supplementary file 1 — ap4c04020_si_001.pdf [file ap4c04020_si_001.pdf]

Supporting Information

De novo amyloid peptide – polymer blends with enhanced mechanical and biological properties

Xianjun Wang[1], Malay Mondal[2], Penelope E. Jankoski[1], Lisa K. Kemp[1], Tristan D. Clemons[1,3], Vijayaraghavan Rangachari[2,3,], Sarah E. Morgan[1,*]*

[1] School of Polymer Science and Engineering, University of Southern Mississippi, Hattiesburg, MS, 39406, USA. [2]Department of Chemistry and Biochemistry, School of Mathematics and Natural Sciences, University of Southern Mississippi, Hattiesburg, MS, 39406, USA. [3] Center for Molecular and Cellular Biosciences, University of Southern Mississippi, Hattiesburg, MS, 39406, USA.

*Corresponding authors: vijay.rangachari@usm.edu; sarah.morgan@usm.edu.

This PDF file includes:

Supplementary Figure 1 to 10

Supplementary Table 1

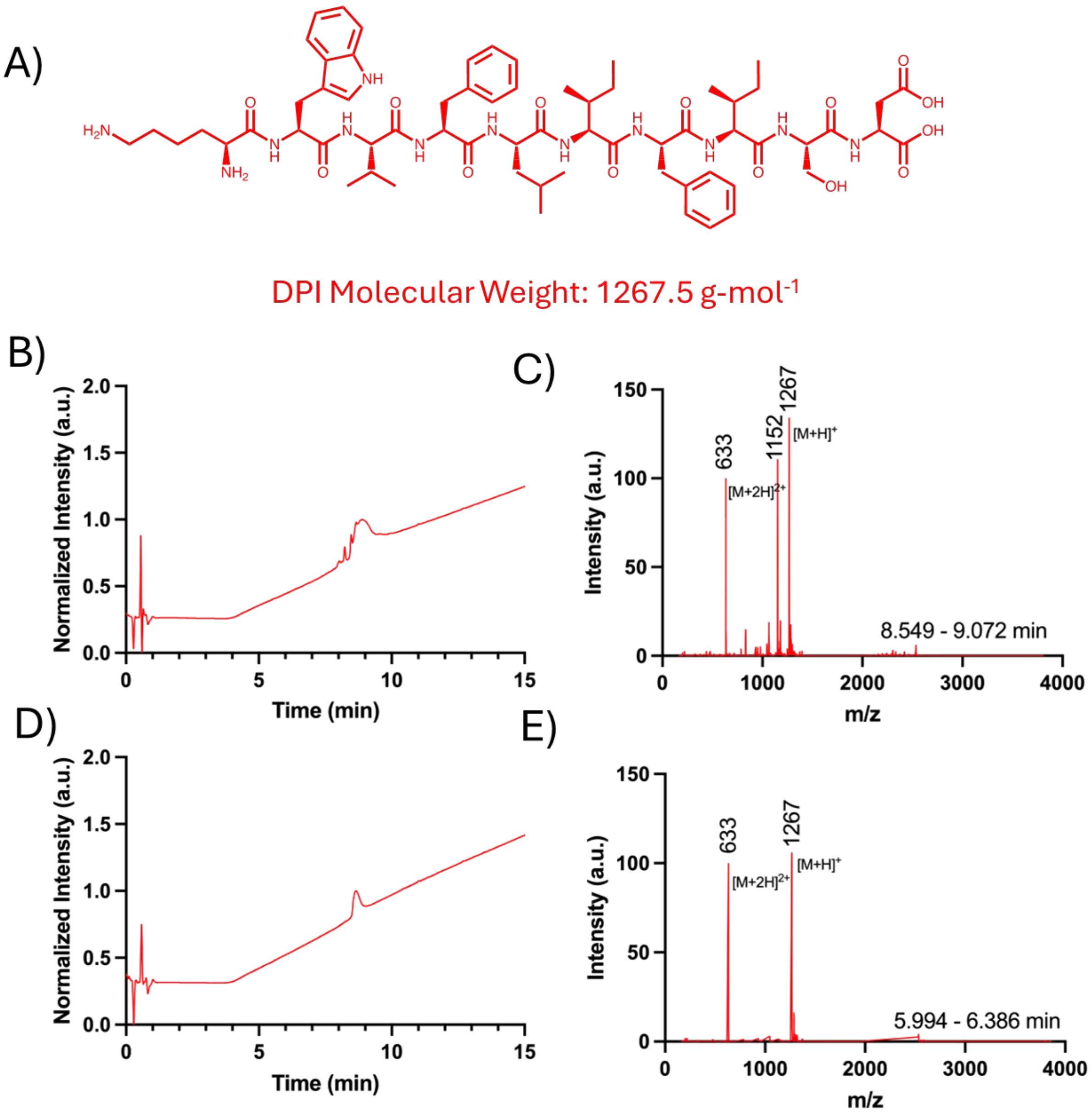

Figure S 1 A) chemical structure and molecular weight of DPI. B) LC-MS trace of crude DPI and C) corresponding crude ESI-mass spectra. D) LC-MS trace of pure DPI and E) corresponding pure ESI-mass spectra. LC-MS conditions: [peptide] = 1 mg/mL, loading solvent; H_2O with 0.1% TFA

(v/v), eluent; H_2O-CH_3CN gradient containing 0.1% HCOOH (v/v), column; Phenomenex Gemini 5 µm C18 110 Å LC column 150 x 1 mm. DP I is approximately 75% pure.

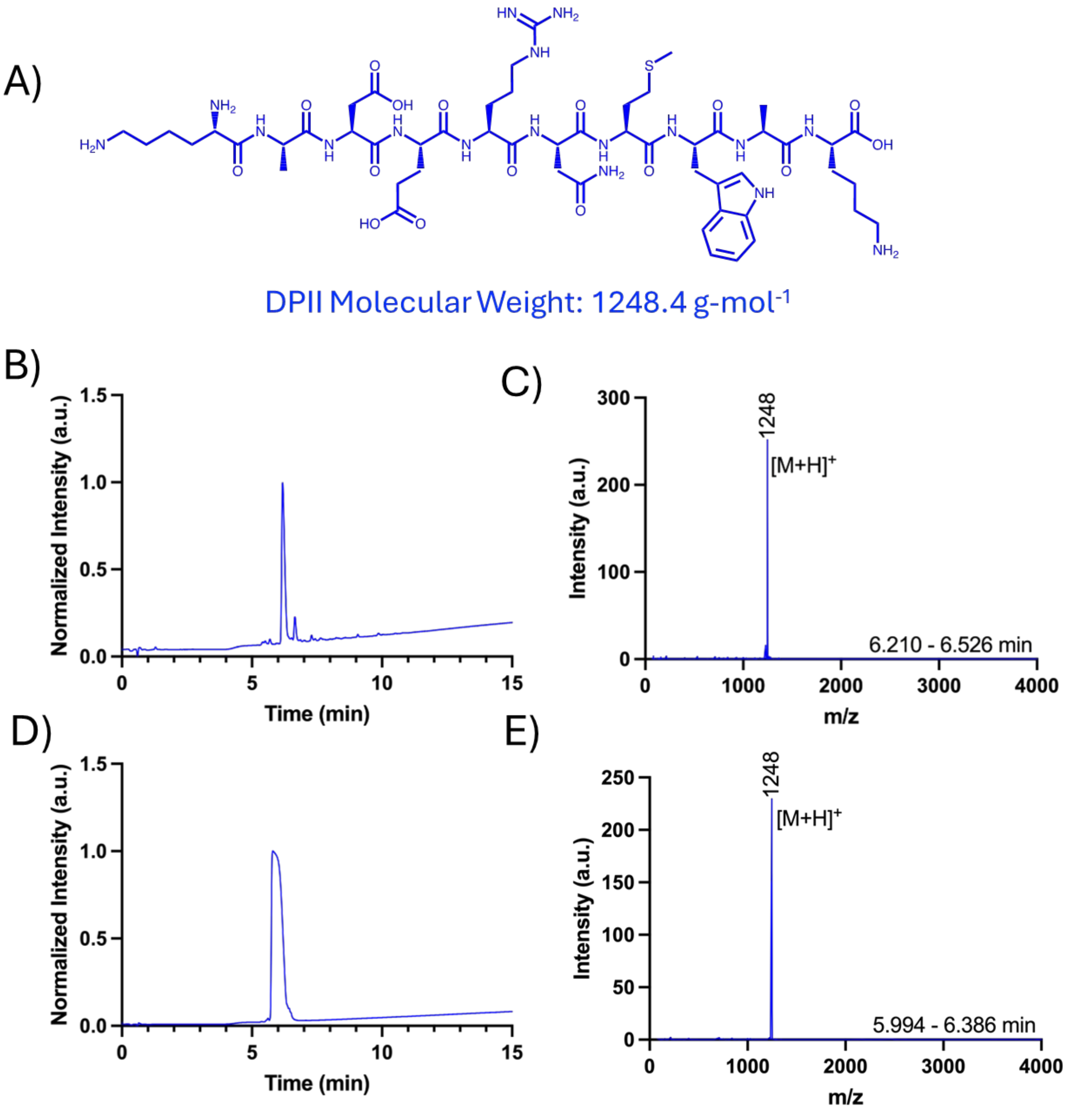

Figure S 2 A) chemical structure and molecular weight of DPII. B) LC-MS trace of crude DPII and C) corresponding crude ESI-mass spectra. D) LC-MS trace of pure DPII and E) corresponding pure ESI-mass spectra. LC-MS conditions: [peptide] = 1 mg/mL, loading solvent; H_2O with 0.1%

TFA (v/v), eluent; H_2O-CH_3CN gradient containing 0.1% HCOOH (v/v), column; Phenomenex Gemini 5 µm C18 110 Å LC column 150 x 1 mm. DP II is approximately 90% pure.

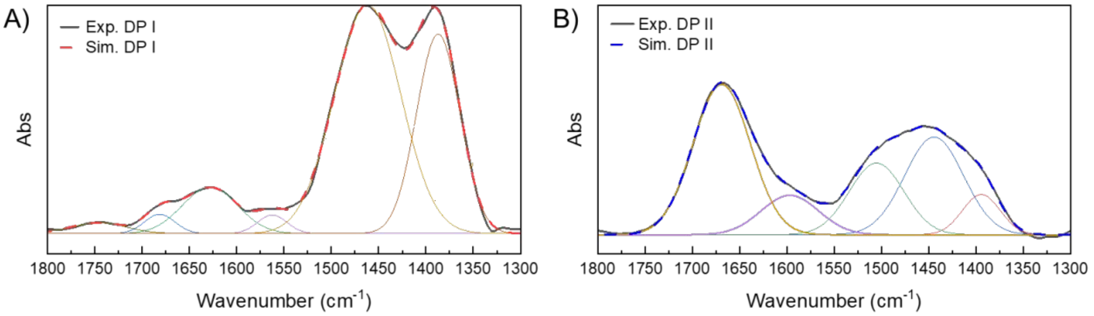

Figure S 3 FTIR spectra of A) DP I and B) DP II. The solid line represents experimental data and the dashed line is the fitted result based on peak deconvolution.

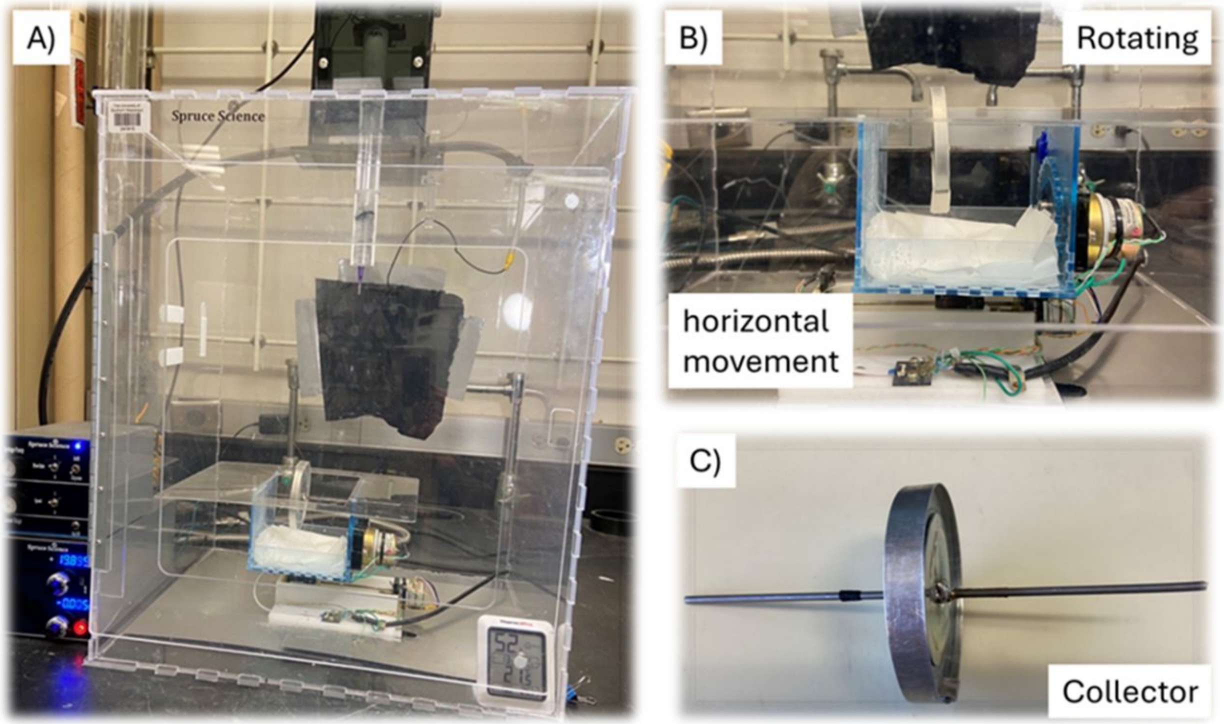

Figure S 4 A) Electrospinning setup. B) The collecting station can move horizontally while the collector rotates. C) A picture of the home-built collector. The collector will move horizontally and rotate simultaneously during collection.

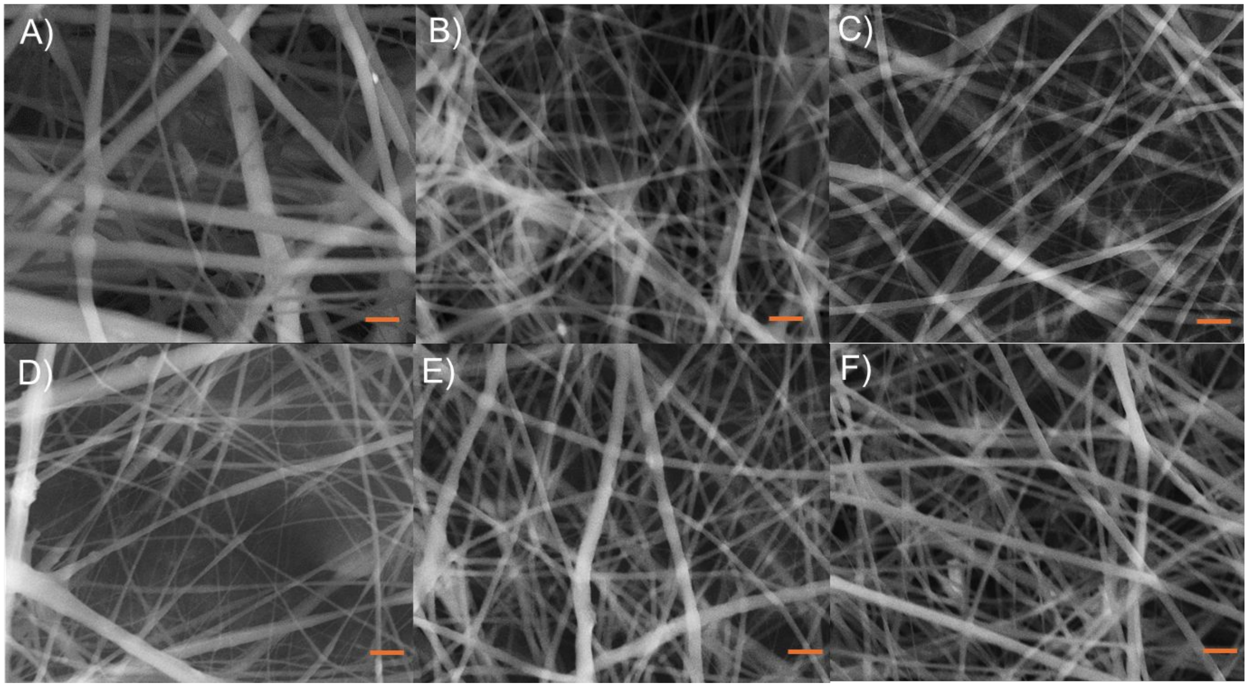

Figure S 5 Additional SEM images of electrospun mats. A) 3-I-1.25; B) 3-I-2.5; C) 3-I-5; D) 3-II-1.25; E) 3-II-2.5 and F) 3-II-5. All scale bars = 2 μm.

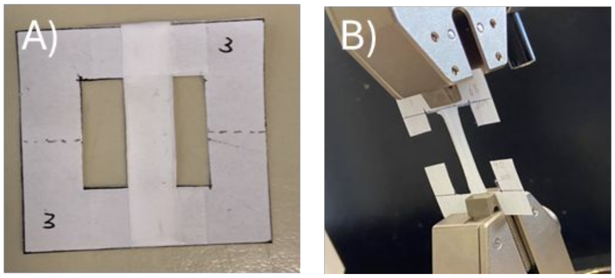

Figure S 6 A) The paper frame is used to prepare tensile bars. B) A snapshot of the tensile test.

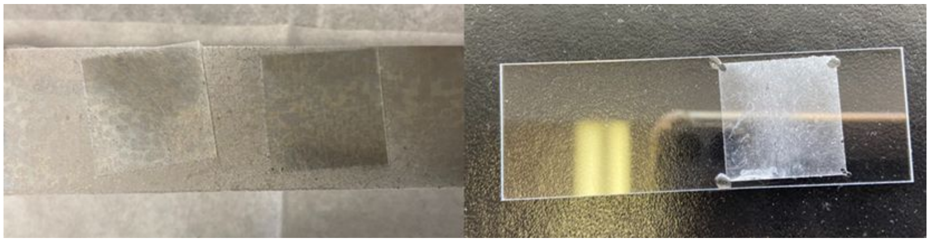

Figure S 7 Aluminum/glass cover as collector (left) and sandwiched sample for imaging (right).

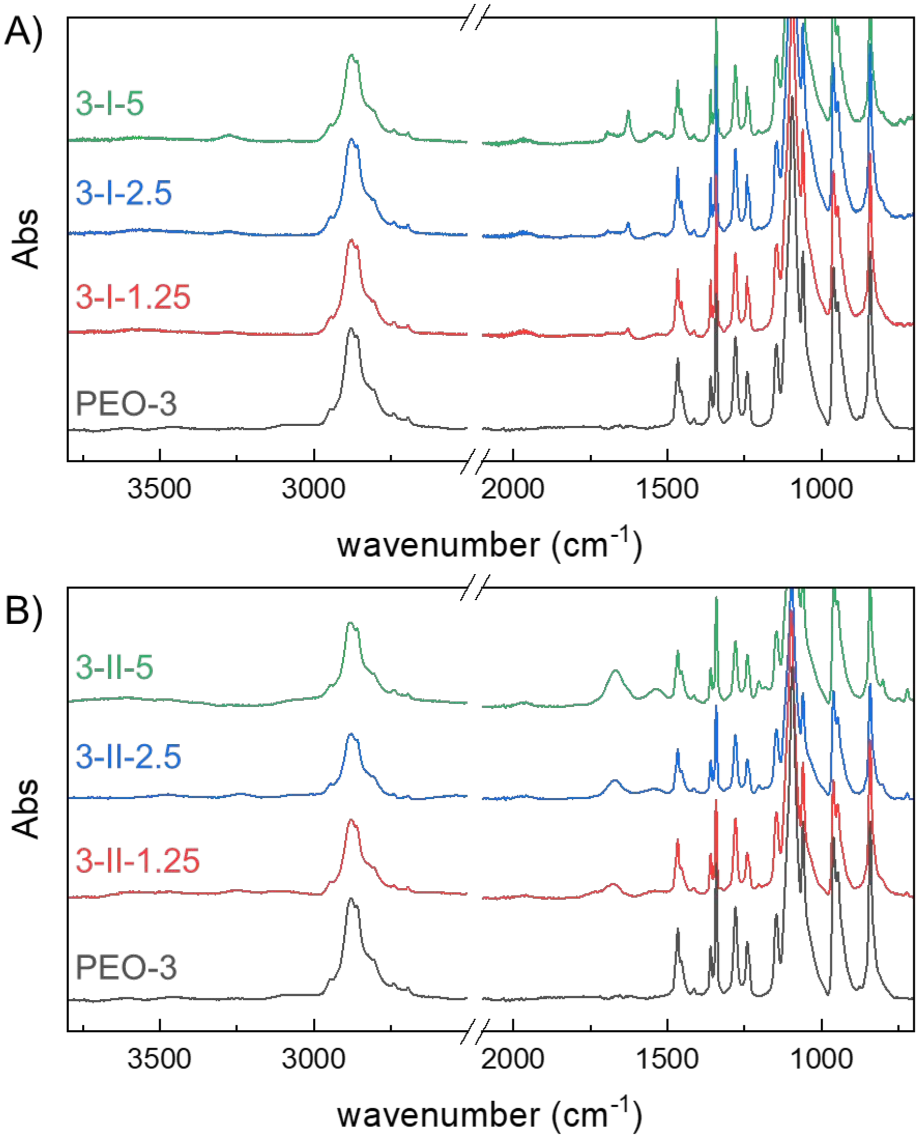

Figure S 8 ATR-FTIR of A) PEO/DP I and B) PEO/DP II composite mats from 3800 to 700 wavenumber.

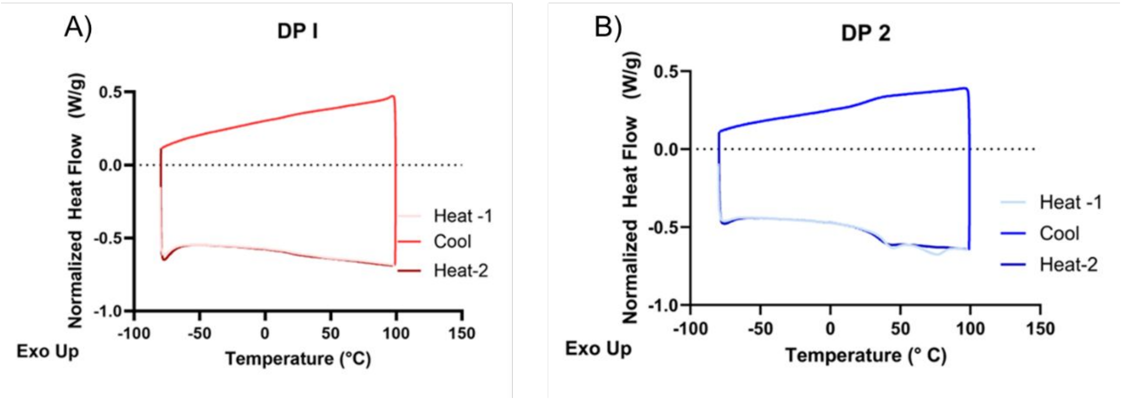

Figure S 9 DSC traces of self-assembled DP I (A) and DP 2 (B) show no thermal transitions on 2nd heating from -80 to 100 °C. There are no T_m or T_c observed in the neat self-assembled peptide samples, as evidenced by the lack of distinct peaks in the cool or second heat. This is frequently seen in short peptides as there are insufficient structural features to yield these thermal transitions.[1] Furthermore, the aggregation into their structures may impede the ability to visualize transitions at lower temperatures. DP II displayed two endothermic peaks in the first heat run that did not correspond to any observable transitions in the subsequent cooling and heating run. This suggests that there is a disintegration or disaggregation event that disrupts the self-assembly of these materials at this temperature. DP I may be more thermally stable and would require more heat to obtain the same transition.

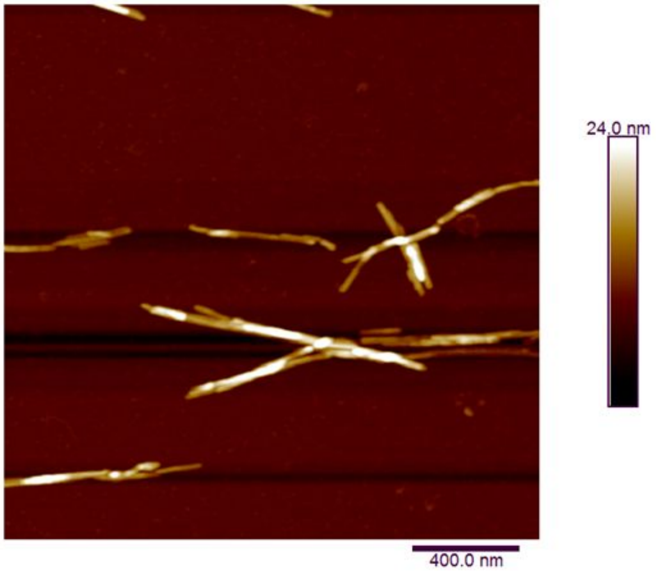

Figure S 10 AFM height image of DP I fibril with a scale bar of 400 nm.

Table S1 Conductivity of DP solutions.

	DP I solution (μS/cm)	DP II solution (μS/cm)
5.00 mg/mL	269.8 ± 1.1	292.1 ± 2.4
2.50 mg/mL	139.6 ± 0.5	184.6 ± 2.4
1.25 mg/mL	78.8 ± 0.2	111.2 ± 1.1

Reference:

1. Ryu, J.; Park, C. B., High stability of self-assembled peptide nanowires against thermal, chemical, and proteolytic attacks. *Biotechnology and Bioengineering* **2010,** *105* (2), 221-230.
